# Indonesian National Health Insurance scheme longitudinal sample data 2015–2020: overview and potential uses for health policy analysis

**DOI:** 10.1186/s12913-025-13756-9

**Published:** 2025-12-12

**Authors:** Alfariany Fatimah, Laura Anselmi, Jonathan Gibson, Asri Maharani, Irmansyah Irmansyah, Sri Idaiani, Dwidjo Susilo, Jack Wilkinson, Matt Sutton, Herni Susanti, Helen Brooks, Penny Bee, Hasbullah Thabrany

**Affiliations:** 1 Health Organisation, Policy and Economics (HOPE), School of Health Sciences, The University of Manchester, Manchester, UK; 2 Mental Health Research Group, Division of Nursing, Midwifery and Social Work, School of Health Sciences, University of Manchester and Manchester Academic Health Science Centre (MAHSC), Manchester, UK; 3 Research Center for Public Health and Nutrition, National Research and Innovation Agency, Cibinong, Indonesia; 4 Research Center for Preclinical and Clinical Medicine, National Research and Innovation Agency, Cibinong, Indonesia; 5 Department of Management and Public Policy, Faculty of Social and Political Sciences, Universitas Gadjah Mada, Yogyakarta, Indonesia; 6 Centre for Biostatistics, Manchester Academic Health Science Centre, Division of Population Health, Health Services Research and Primary Care, University of Manchester, Manchester, UK; 7 Mental Health and Psychiatric Nursing Cluster, Faculty of Nursing, Universitas Indonesia, Depok, Indonesia; 8 Kalta Bina Insani (KBI) Consulting and Training, Jakarta, Indonesia

**Keywords:** BPJS kesehatan sample data, Indonesian National Health Insurance, Health administrative data

## Abstract

**Background:**

The Indonesian National Health Insurance Agency (BPJS-K) administers one of the largest single-payer healthcare systems in the world, with 95% of Indonesia’s population registered by December 2023. Since 2019, BPJS-K has provided sample data representing 1% of insured individuals. Despite its potential, the BPJS-K sample data remains underutilised in research.

**Methods:**

This study provides an overview of the BPJS-K dataset, including research that has used it, its structure, contents, and key variables from 2015 to 2020. We present descriptive statistics for the sample, including age and gender distributions, insurance membership type, healthcare visits, diagnoses, referrals, and associated tariffs. We illustrate the dataset’s potential applications for health policy analysis and its strengths and limitations.

**Results:**

The BPJS-K sample data broadly represents the Indonesian population, as evidenced by comparisons to census data. Regional disparities in healthcare utilisation were observed, with lower service access in Eastern Indonesia. Key variables include diagnoses, such as acute respiratory infections (6% of the visits, the highest in primary healthcare), and reimbursement information for visits to referral healthcare providers and for non-capitation services to primary healthcare providers. The data has the potential to facilitate health policy analysis, given its longitudinal nature and possible linkage to other data. However, current shortcomings, such as limited socio-economic information and quality of diagnostic information, should be considered.

**Conclusion:**

The BPJS-K sample data offers potential for longitudinal and cross-sectional health policy research. However, further improvements in data quality, diagnostic recording, accessibility, and linkage to socio-economic data are recommended to optimise its usefulness for research.

**Supplementary Information:**

The online version contains supplementary material available at 10.1186/s12913-025-13756-9.

## Background

The Indonesian government’s effort to provide accessible health services has evolved from a fragmented health insurance system (1945–2013), which primarily served the military and civil servants, to universal health coverage through the establishment of the National Health Insurance Agency or *Badan Penyelenggara Jaminan Sosial Kesehatan (BPJS-K)* (Law 40/2004 and Law 24/2011) [1]. In 2014, the BPJS-K introduced the National Health Insurance or *Jaminan Kesehatan Nasional (JKN)* and has since actively expanded coverage to 267.3 million people, approximately 95% of Indonesia’s population, by December 2023 [2]. The BPJS-K is the sole institution managing its members’ health administrative data, resulting in the largest single-payer insurance dataset globally [1]. As of 2021, it manages 50 billion patients’ records nationally [3]. Since 2019, it has provided sample data representing one per cent of its members, although the data remains underutilised in health research despite its potential.

Few studies have used this data, and those that have provide limited explanations of the data structure, sampling, and variables that could be helpful to other data users. Although the BPJS*-K* provides data guidance in *Bahasa Indonesia* [3–5], no internationally accessible description exists. This study addresses that gap by providing a comprehensive overview of the data to support wider use for in health policy analysis and design.

This paper outlines the structure of BPJS-K sample data, the sampling method, and presents descriptive analyses of key variables. It shows the representativeness of the sample to the Indonesian population, variation in membership distribution, membership status, healthcare utilisation and recorded diagnoses, referral, and claimed cost. It discusses its strengths, limitations of the data, and guidance for future use.

### The Indonesian National health Insurance program

#### Revenue collection and registration

BPJS-K revenue primarily comes from membership contributions (Presidential Regulation (PR) No.82/2018), supplemented by BPJS-K investments, government funding, and tobacco tax (since 2018) [6]. To achieve universal health coverage (Law No. 40/2004), the government incentivises registration by requiring BPJS-K membership to access essential public services, such as school registration (Presidential Instruction No.1/2022) and driving licenses (Indonesian Police Regulation No.2/2023). Moreover, the government pays the contributions for poor individuals listed in the Integrated Data of Social Welfare, whose eligibility is assessed by the district government and agreed upon by the Ministry of Social Affairs, reviewed every two years (Law No.13/2011), and verified and validated by the Indonesian Central Bureau of Statistics.

The BPJS-K registration is household-based requiring all individuals listed on the family card to be registered simultaneously, except those living abroad (BPJS-K Regulation No.5/2020). When an individual no longer resides but still listed in the family card, for example students, institutionalised individuals or those in temporary housing, their registration remains linked to the family card.

Membership is categorised by contribution source: non-workers (e.g., employers, pensioners, and war veterans), salaried workers, non-salaried workers (e.g., informal workers, farmers, and freelancers), central-government-supported and local-government-supported members. Inpatient class is determined by contribution level and affects room standards, though medical services are uniform. Room standards vary in terms of type, facilities, and occupancy level. For example, Class 1 typically accommodates 1–2 patients, Class 2: 3–5 patients, and Class 3: 4–6 patients. The government plans to standardise hospital wards by 2025 to ensure equitable services regardless of their financial contribution (PR No.82/2018 and PR No.59/2024).

BPJS-K membership contributions and class assignments are regulated as follows: salaried workers contribute 5% of their salary (4% employer, 1% employee), The contributions capped at IDR 12 million (USD 750), with Class 1 assigned to those earning above IDR 4 million (around USD 250) or otherwise Class 2. Laid-off salaried workers may access Class 3 without a contribution payment for six months (PR No.59/2024). Non-salaried workers and non-workers can choose their class by paying different fixed monthly contributions, which increased in 2021 from IDR 25,500 to 35,000 (USD 1.57–2.16) for Class 3, IDR 51,000 to 100,000 (USD 3.14–6.15) for Class 2, and IDR 80,000 to 150,000 (USD 4.92–9.23) for Class 1 (PR No.64/2020). The government subsidies for Class 3 also decreased from IDR 16,500 to 7,000 (USD 1.02–0.43). Government-supported individuals are only eligible for Class 3 (IDR 42,000 or USD 2.58 paid by the government).

When a member fails to regularly pay monthly contributions, they become inactive members, and to become an active member again, lapsed contributions must be paid. An inactive member can access care but would be personally responsible for the cost. If using care within 45 days of reactivating BPJS-K membership, a co-payment is expected, 2.5% of the cost up to a maximum of IDR 30 million or about USD 1,840 (PR No.82/2018), increased to 5% in 2020 (PR No.64/2020 & PR No.59/2024).

#### Resource pooling

BPJS-K operates under a single pooling system where all members share financial risk regardless of health status. BPJS-K manages the fund nationally. Continuously expanding membership and increasing contribution compliance are supported by introducing an initiative such as corporate social responsibility-based crowdfunding to support individuals facing financial hardship in paying contributions [7].

#### Services purchasing and provision

BPJS-K is responsible for purchasing and paying for healthcare services, whilst the government regulates the scope of services (PR No.82/2018 and PR No.59/2024) and the tariffs for healthcare providers through the Ministry of Health Regulations. The scheme covers primary care (PHC) and referral care (RHC), including specialist and sub-specialist care (PR No.82/2018), and selected health screenings (PR 59/2024). BPJS-K contracted both government-owned and private providers to deliver these services (PR No.82/2018).

PHC services under BPJS-K are funded through both capitation and non-capitation payments. Capitation services include non-specialist diagnoses, which are regulated in the practical guidance (HMR No.5/2014) and the general practitioners competency standard (Indonesian Medical Council No.11/2012). Capitation payments are made in advance per registered patients (Health Minister Regulation (HMR) No.69/2013, No.59/2014, No.52/2016, No.3/2023). Since 2015, performance-based capitation payments have been applied based on contact rates, non-specialist referral ratio, and chronic patients measured by the visits ratio of people in *PROLANIS*, a program aimed at improving care for hypertension and/or diabetes patients (Ministry of Health and BPJS-K regulation No.3/2016, BPJS-K Regulation No.2/2015 and No.7/2019). Current regulations also adjust payments by national age-sex-based health risk, with special capitation tariffs for remote areas (HMR No.3/2023). BPJS-K may redistribute member registrations among PHCs (BPJS-K regulation No.1/2017) based on provider capacity, measured by the ratio of doctors and dentists to BPJS-K members (Ministry of Health No. HK.01.07/MENKES/2194/2023). The utilisation of capitation payments in government-run PHCs is regulated in more detail in HMR No.6/2022, including the remuneration system.

Non-capitation services are reimbursed on a fee-for-service basis, and claims must be submitted by the PHC and verified by the BPJS-K before reimbursement as regulated by the Health Minister Regulation (HMR) No.3/2023 and the BPJS-K Regulation No.7/2018. These include ambulance transfers; maternal, neonatal and contraceptive services; chronic disease monitoring, such as medicine microalbuminuria and blood tests; cryotherapy for cervical cancer; inpatient care; dental prostheses; health screenings for cervical cancer, diabetes mellitus, thalassemia, and colon cancer; and emergency services at non-contracted PHC.

Referral healthcare (RHC) includes specialist and sub-specialist outpatient and inpatient services, as defined by Presidential Regulation (PR) No. 82/2018 and subsequently maintained in PR No.59/2024. Access requires a referral from primary care providers which is valid for three months from the date in the referral letter but can be re-referred by PHC if needed (cl.55 PR No.82/2018). RHC services are reimbursed using the Indonesian Case-mix-Based Groups (INA-CBG) tariff (The Health Minister Regulation no. 52/2016), a case-mix-based system, with claims submitted by providers and verified by BPJS-K, and non-INA CBG tariffs. The INA-CBG tariff categorises costs by medical diagnosis and procedure and groups them into five regions based on each Province’s Consumer Price Index (CPI), updated to the provincial minimum wages index in Health Minister Regulation No. 3/2023.

#### Changes in regulations and policies

All the policies and regulations refer to Law No. 40/2004 on the National Social Security System and Law No.24/2011 on the Social Security Agency. Recent regulatory changes include updates on membership rules, contribution rates and inpatient ward standards, inclusion of tobacco tax, and emphasis on the health information system. Appendix Fig. 1 shows the timeline of the regulation changes.

#### Health information system

BPJS-K’s Information and Technology Directorate manages the health information system, which includes infrastructure for recording membership data, patient-level visit data and payment claims from contracted providers and regional offices. Data is collected as part of providers’ contractual obligations using either available electronic medical record systems or manual reporting. The BPJS-K ensures data quality, safety and privacy protection.

## Methods

### Dataset structure

In 2019, BPJS-K released pseudo-anonymised sample data covering information for 2015 and 2016, updated biennially then annually after 2020. It is representative from district to national levels, and links administrative and claims data [3–5]. The dataset is longitudinal, tracking existing individuals and adding new individuals to maintain the 1% sample of BPJS-K members, and it captures repeated measures over different periods.

**Fig. 1:**
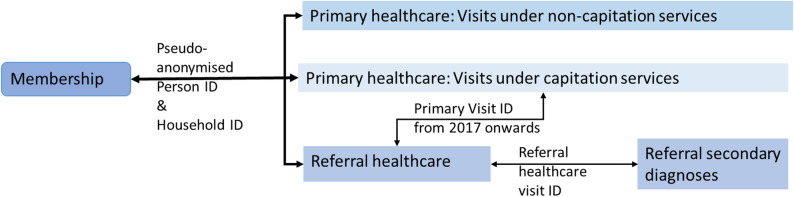
Datasets and linkages. Source: Authors’ interpretation of the datasets

The BPJS-K sample data are organised into five datasets: membership, primary care visits (capitation and non-capitation services), referral care visits and their associated secondary diagnoses. All records are linkable via pseudo-anonymised ID, primary care visits within capitation services are linkable to the respective referral visits, and referral secondary diagnoses are linkable to the corresponding referral visit (Fig. 1).

BPJS-K also provides pseudo-anonymised contextual datasets for diabetes mellitus, tuberculosis, and mothers and children’s health. These share a similar structure, but the sampling frame is drawn from registered members diagnosed with a specific condition or a specific group of people [8], and is not included in our analysis.

### Dataset content

The membership dataset contains detailed information on BPJS-K members, and each row represents a member. The pseudo-anonymous individual and household IDs shows no attrition in the record across the 2015–2020 period (Appendix Table 4).

The membership dataset includes information on (i) members’ demographics (date of birth, sex, marital status, and year of death, where applicable), (ii) province and district of residence, (iii) primary healthcare provider information includes ownership, type, and province and district, (iv) membership segmentation, inpatient class and active status, and (v) the sample weight. A more detailed list of variables is reported in Appendix Table 5. The variables are updated for each data period, but the exact date of the update within the two-year period is not provided. There are members’ transitions between active and inactive status across periods, reflecting their compliance with contribution payments (Appendix Table 6).

Primary healthcare visits datasets comprise visits for both services covered (capitation) and not covered (non-capitation) by capitation payments. Both datasets include details on visits, providers, and diagnoses using ICD-10. The non-capitation dataset has additional treatment details, the provider’s cost claimed, and verified cost by BPJS-K. Non-capitation includes detailed treatment data because services are reimbursed on a fee-for-service basis, with some, such as antenatal care, being reimbursed as a package, and requiring specific documentation. Meanwhile, capitation services lack such details since payments are not tied to individual treatments and data is less often collected or reported. The tariffs for non-capitation treatment claims are determined by the Ministry of Health regulation with the latest being HMR No.3/2023. Appendix Table 10 outlines variables included in both datasets.

Referral healthcare visits datasets and referral secondary diagnoses (details on variables in Appendix Tables 16 and 17). Access to referral healthcare is through referral system from primary care, except in an emergency. The dataset has information on the date and place of the visit, provider details, referring primary care provider, diagnosis (on admission and primary), claimed and verified cost, and the INA-CBGs code. The secondary diagnosis dataset includes only four types of information: referral care visit codes, 3–5-digit ICD-10 codes, 3-digit ICD-10 codes, and the name of the diagnoses. Overall, there are three types of diagnoses recorded: diagnosis at hospital admission, primary diagnosis, and secondary diagnosis. For claim purposes, the INA-CBG code is tied to the primary diagnosis in referral healthcare visits.

### Sampling

The sampling frame includes all BPJS-K members and is stratified based on primary healthcare (PHC) and three household types: [1] households with no recorded PHC visits [2], those with recorded PHC visits, and [3] those with both PHC and RHC visits. In every period in each PHC, including newly established ones, one additional household per stratum was sampled beyond the original 10 households selected in 2015–2016 [3, 5]. All new household members are included in each period. The dataset includes individual and household-level sample weights. Further details on the sampling methodology are provided in Appendix Section A2.

### A systematic search of studies using the BPJS-K sample data

We conducted a systematic search restricted to the period 2015–2023 for studies using BPJS-K sample data across platforms: PubMed, Google Scholar (article), CORE (research), SSRN, MPRA, Science Direct (research), and Taylor & Francis (article). Search terms included a combination of BPJS AND sample data, Indonesian AND National AND Health AND Insurance AND (INHI) AND “sample data”, *Jaminan* AND *Kesehatan* AND *Nasional*, JKN, *Badan* AND *Penjamin* AND *Jaminan* AND *Kesehatan*, “National Health Insurance” AND Indonesia, universal AND health AND coverage AND Indonesia, “Social security agency” AND health AND Indonesia.

### Data analysis

A descriptive analysis assessed representativeness of the BPJS-K sample data by comparing its population structure to the 2020 Indonesian census. We examined sampling representation by province, tracked membership status changes over the years, and examined variations in healthcare utilisation across provider types and geographic areas in Indonesia. We described common diagnoses in primary and referral healthcare, and the costs claimed by providers in referral healthcare.

## Results

### Studies using the BPJS-K sample data

Out of 6,712 studies retrieved (Appendix Table 1), we retained 12 studies that used BPJS-K sample data (Appendix Table 2) and six studies that used BPJS-K data from the 2014–2017 claims report [9] (Appendix Table 3). Our review focused on BPJS-K members who accessed BPJS-K contracted healthcare providers. All but one of the retained studies using BPJS-K sample data are published in peer-reviewed journals and cover a range of topics. Nine focused on a specific illness, conditions, and/or procedures, including pre-eclampsia [10], dengue fever [11], chronic disease [12], cancer [13], diabetes [14], hypertension [15, 16], haemodialysis [17], and HIV [18]. These studies descriptively examine service utilisation [13–15, 17–20], condition-related costs [16], and referral patterns for specific illnesses [11, 13]. Other studies discuss referral compliance and health-seeking behaviour [21], the impact of performance-based capitation payment on PHC utilisation [20], and the relationship between temperature and non-communicable disease [22].

Most studies used the 2015–2016 sample data, and only two used the 2018–2019 dataset [13, 18]. Two studies combined the dataset with village potential (PODES) [21] and survey data from Statistics Indonesia (BPS) at the provincial level [13]. Although most analyses were conducted at the individual-level, four studies used aggregated district- or provincial-level data [13, 20–22], enabling integration with other datasets and expanding the analysis. The BPJS-K sample data was used for risk prediction models [10], policy evaluation [20], and analysis of patient characteristics and diagnostic patterns [12–15, 17, 18]. Methods applied to the data ranged from descriptive analysis to multivariate regression and causal inference techniques, such as difference-in-differences.

### Sample description

In the 2015–2016 period, the BPJS-K sample data included 1.6 million individuals (about 1% of total members) across 586,969 households (Table 1). These were sampled from a population of 171 million members in 73 million households registered with 22,024 primary healthcare facilities. By 2020, the sample had grown to 2.2 million individuals, maintaining 1% coverage.

**Table 1:** BPJS-K total members and sample datasets individuals, 2015–2020

	Year				
	2015–2016^	2017	2018	2019	2020
Members* (Newly registered)	171,939,254	187,982,949 (+ 16,043,695)	222,002,996 (+ 34,020,047)	224,149,019 (+ 2,146,023)	222,461,906 (-1,687,113)^^
Registered households*	73,441,160	81,068,954	89,469,411	101,919,290	107,298,546
(Newly registered)		(+ 7,627,794)	(+ 8,400,457)	(+ 12,449,879)	(+ 5,379,256)
Population of PHC** (Newly PHCs)	22,024	21,969 (− 55)		22,927 (+ 958)	
**Samples**					
Household sample (Newly sampled)	586,969	643,760 (+ 56,791)	703,924 (+ 60,164)	764,531 (+ 60,607)	822,842 (+ 58,311)
Individual sample (Newly sampled)	1,697,452	1,832,418 (+ 134,966)	1,971,744 (+ 139,326)	2,093,156 (+ 121,412)	2,200,960 (+ 107,804)

Notes:

*The number of registered members and households is based on BPJS-K’s public financial report, which captures the number as of December each year [6]. For the 2015–2016 period, this refers to December 2016

** The PHCs figure is sourced from the BPJS-K sample data guidebook and may differ from those in the BPJS-K financial report [3–5].

^ 2015 and 2016 period serves as the base year for sampling, using the numbers of PHCs, registered members and households at some point in that period. BPJS-K uses the same number of PHCs for each two-year period. The total number of registered households (from BPJS financial report [6]) formed the sampling frame for the base year, and the newly registered households were added in each subsequent year

^^ This figure refers to the overall BPJS system and is derived from administrative records that monitor enrolment and deregistration, as published in the BPJS-K public financial report [6]. Deregistration is identified when members fail to meet eligibility criteria, such as non-payment of premiums or expiration of coverage periods. The decline in the number of deregistered individuals in 2020 may be due to the COVID-19 pandemic, which led to members’ deaths and voluntary deregistration, or data cleansing of government-assisted members [3]. Additionally, an increase in the BPJS-K contribution from January to March 2020 may have contributed to deregistration. In October 2019, the Supreme Court cancelled Article 34 of Presidential Regulation No.75/2019 and reinstated the previous tariff [23]

Source: BPJS-K Data Portal [8] and BPJS-K sample data book [3–5]

The sample distribution is concentrated on Java Island, consistent with 60% of Indonesians residing there (Appendix Figure A2.1). However, over-representation occurred in Papua and Maluku regions, South Sulawesi Province, and Bengkulu Province in Sumatra Island, and under-representation in several provinces in Java Island, West Nusa Tenggara Province and Aceh Province (Appendix Figure A2.1).

### Sample demographics

**Fig. 2:**
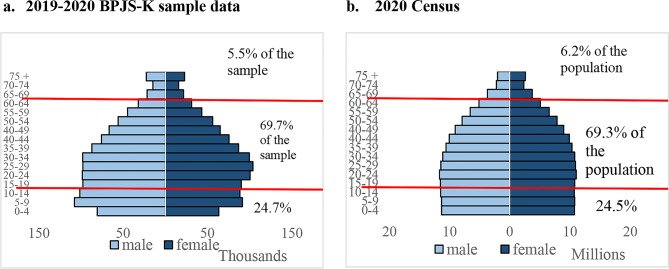
Distribution by age and sex. Note: Horizontal lines at 15 and 65 to highlight working age population. Source: BPJS-K sample data 2015–2020, author calculation. Census 2020, BPS [23]

The BPJS-K sample data closely mirrors the national population structure, with the working-age population (15–64 years old) comprising 69.7% compared to 69.3% in the 2020 Census (Fig. 2). A Chi-square test of both population structure showed no significant differences between the BPJS-K sample data and census data (Appendix Table 8). A slight underrepresentation of older adults (0.7%) in the sample data may reflect lower enrolment among this age group. A small proportion of individuals (0.008%) were misreported as deceased in 2017–2018 but reappeared as active or inactive members in 2019–2020 (Appendix Table 6).

### Membership status

In 2019–2020, 41% of individuals were receiving central or local government assistance (Table 2). Local governments support rising from 5% in 2015–2016 to 9% in 2019–2020, easing some central government’s burden. Salaried workers in the inpatient Class 1 increased by 3% points, whilst non-salaried workers were mainly in inpatient Class 3 (15% in 2019–2020), indicating a lower ability to pay.

**Table 2:** BPJS-K registered members by inpatient ward class and segmentation

Inpatient Class	Segmentation	2015–2016		2017–2018		2019–2020	
		Individuals	%	Individuals	%	Individuals	%
Class 1	Non-Workers	38,031	2.24	41,545	2.11	47,097	2.14
	Non-salaried Workers	53,258	3.14	62,332	3.16	51,488	2.34
	Salaried Workers	207,866	12.25	238,796	12.11	338,372	15.37
Class 2	Non-Workers	27,007	1.59	28,555	1.45	31,552	1.43
	Non-salaried Workers	59,139	3.48	89,830	4.56	75,684	3.44
	Salaried Workers	452,089	26.63	469,919	23.83	419,908	19.08
Class 3	Non-Workers	483	0.03	1,190	0.06	1,782	0.08
	Non-salaried Workers	134,728	7.94	255,189	12.94	325,835	14.80
	Salaried Workers	149	0.01	334	0.02	136	0.01
	Central Govt. Assistance (PBI APBN)	633,943	37.35	640,073	32.46	706,218	32.09
	Local govt. Assistance (PBI APBD)	85,857	5.06	140,182	7.11	201,615	9.16
Missing		4,902	0.29	3,799	0.19	1,273	0.05
Total		1,697,452	100	1,971,744	100	2,200,960	100

Note: The membership dataset information was updated in a two-year period: 2015–2016, 2017–2018, and 2019–2020, hence the two-year period in the table

### Healthcare utilisation

Between 2015 and 2020, there were 1,227,290 individuals (56% of total BPJS-K members in 2019–2020) who made 11,122,708 primary healthcare capitation services visits (Table 3). In contrast, non-capitation services were used by 101,220 individuals (5%), accounting for 406,320 visits. Referral healthcare was accessed by 619,750 individuals (28%) who made 4,268,184 visits (Table 3).

Primary healthcare visits within capitation services had the highest utilisation of 1,200-3,000 per 10,000 BPJS-K members (Table 3). Only 85–100 individuals used non-capitation services among 10,000 BPJS-K members (Table 3). Referral care was accessed by 600–700 individuals per 10,000 BPJS-K members, lower than primary care, as expected under the referral system.

**Table 3:** Healthcare utilisation by type of healthcare and year

Visit year	Total BPJS-K members	Primary healthcare providers				Referral healthcare providers	
		Capitation services		Non-capitation services			
		The mean number of visits per individual	Rate per 10,000 members who made at least one healthcare visit	The mean number of visits per individual	Rate per 10,000 members who made at least one healthcare visit	The mean number of visits per individual	Rate per 10,000 members who made at least one healthcare visit
2015	156,790,287	2.78	1259	2.40	85	3.29	683
2016	171,939,254	2.96	1573	2.45	104	3.42	725
2017	187,982,949	3.38	3044	2.92	87	4.46	752
2018	222,002,996	3.70	2944	3.17	85	4.72	727
2019	224,149,019	3.92	3278	3.04	98	4.88	766
2020	222,461,906	3.92	2843	2.64	100	4.87	600

Note:

The mean number of visits was calculated by dividing the total number of visits (weighted by sample weight) over the total individuals (also weighted by sample weight) who had at least one visit, more details in Appendix Tables 11, 13 and 18

Primary healthcare visits include both healthy visits and sick visits (FKP22) and not available in the non-capitation dataset. The healthy visit is mainly for health promotion and disease prevention coded as 9999 with no diagnosis information recorded

### Primary care

Between 2015 and 2020, approximately 37%-39% of individuals exist in the membership dataset but do not have any records in the visit datasets, consistent with the sampling design (Appendix Table 9). In comparison the National Socioeconomic Survey’s (SUSENAS) percentages of households reporting access to primary healthcare were 26% in 2019 and 23% in 2020 [24].

**Fig. 3:**
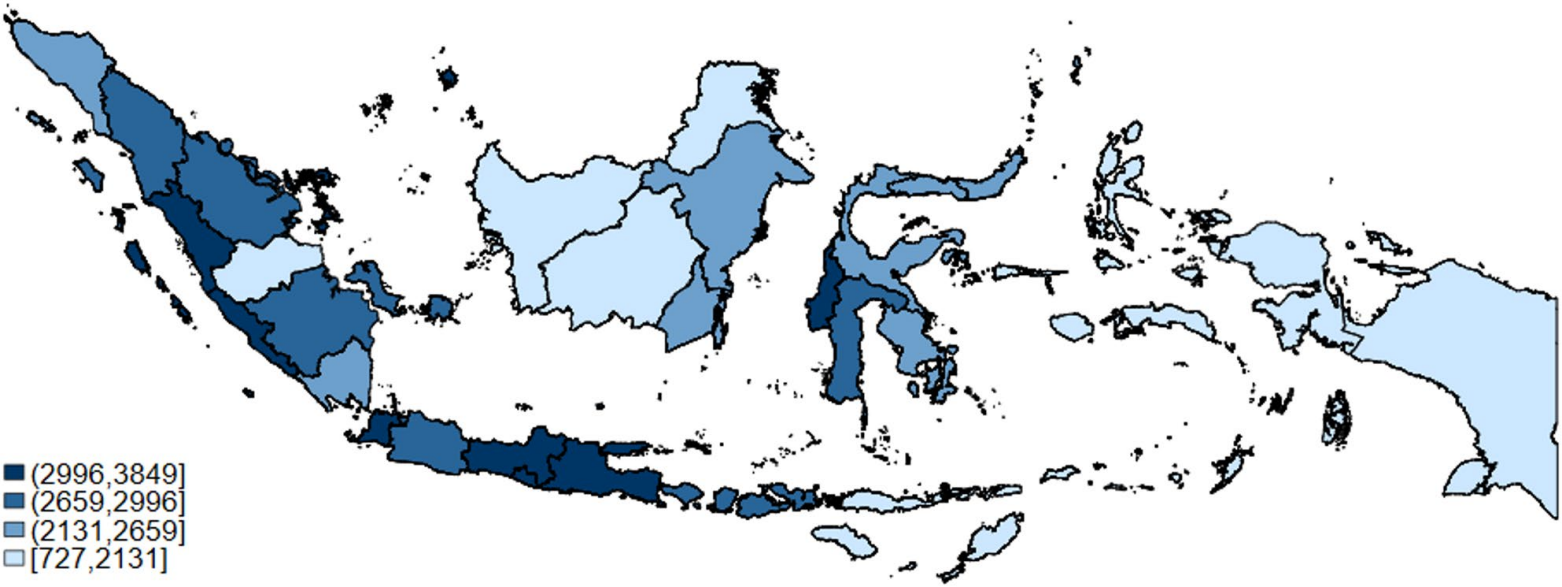
Rate of weighted individuals visited PHC per 10,000 BPJS-K members by Province, 2020. Source: Authors’ calculation. The BPJS-K registered members data by province obtained from the National Insurance Board (DJSN) website. Notes: The black line in the figure represents provincial boundaries

Figure 3 shows comparisons across district of the weighted individuals who visited primary healthcare at least once in 2020 per 10,000 total BPJS-K members. We found a concentration in Java Island, where each province had 3,000–3,300 per 10,000 BPJS-K members. The provinces on Sumatra Island had more variation, with Jambi having the lowest rate (2,000 per 10,000 BPJS-K members), and West Sumatra and Bengkulu (3,000 and 3,300 per 10,000 BPJS-K members) having the highest rate. In Sulawesi Island, most provinces had similar rates (around 2,500-2,600 per 10,000 BPJS-K members), except for a higher rate in West Sulawesi and South Sulawesi (3,300 and 2,800 per 10,000 BPJS-K members). Provinces in Kalimantan Island, Maluku Archipelago, Papua Island, and East Nusa Tenggara Archipelago had the lowest rate (around 700-2,100 per 10,000 BPJS-K members) in 2020, suggesting limited access compared to Java, Sumatra, and Sulawesi. These provincial differences, based on quartile grouping, were consistent across 2015–2019, but 2020 rates are lowest, most likely due to the shock from COVID-19 (Appendix Fig. 2).

Between 2015 and 2020, there were 12,778 diagnoses, with about 48% recorded in fewer than ten visits (Appendix Fig. 3). The most common diagnosis in primary care capitation services was acute upper respiratory infection, unspecified (J069), which is about 650,000 or 6% of all visits within capitation services (Appendix Fig. 4), compared to 4.4% prevalence from the basic health survey (RISKESDAS). About 30% of visits within capitation services were visits for health promotion and disease prevention services, primarily delivered by PUSKESMAS (78% of healthy visits) (Appendix Table 12).

We observed changes in visit numbers for some treatments in non-capitation services, potentially reflecting shifts in BPJS-K regulations. For example, blood cholesterol tests increased from none in 2015 to 29% (around 23,000 visits) in 2020 (Appendix Table 14), likely due to the inclusion of blood tests for patients referred back to primary care under BPJS-K Regulation No.7/2018. BPJS-K potentially re-categorised treatment types as inpatient admissions accounted for 51% of non-capitation visits in 2015, reducing to 34% in 2016 and only 1%-4% in the subsequent years (Appendix Table 14). This shift altered the cost distribution across treatment types, where vaginal birth became the highest after 2016 (Appendix Table 14).

### Referral care

We found 85% of referral care visits are outpatient, and 2.7 million visits (75% of total outpatient visits) had a corresponding ICD-10 code Z00-Z99 (factors influencing health status and visits with health services) (Appendix Table 19). This includes follow-up examinations after treatments for other conditions (34% of referral outpatient visits), occupational and physical therapy, palliative care, special screening, and others. There were 8,446 distinct primary diagnoses and 53% had fewer than ten visits during 2015–2020 (Appendix Fig. 5). Between 2015 and 2020, about 79% of total referral care visits included secondary diagnoses, where diseases of the circulatory system being the most common (18% of visits with secondary diagnosis) (Appendix Table 20).

Referral visits were highest in Java (Regional 1) and lowest in Regional 5 (4%) but the highest mean tariff per visit, reflecting its remote and challenging access (Table 4). Inpatient admissions were fewer but had a higher mean tariff than outpatient visits, resulting in a much higher mean tariff for each admission (about IDR 4 million) than for outpatient (about IDR 300,000).

**Table 4:** Referral healthcare provider (RHC) visits and top-up payment and cost claimed by INA-CBG region 2015–2020

INA-CBG Regions	Total visits	% visits	INACBGs Tariff (IDR billion)	Mean Tariff per visit (in IDR)	INA-CBGs top-up tariff (IDR billion)					Claimed by RHC (IDR billion)	Verified by BPJS (IDR billion)
					SA	SP	RR	SI	SD		
**Total**											
REGIONAL 1	2,414,945	57%	2,090	865,444	0.12	39.60	5.62	3.15	17.8	2,160	2,160
REGIONAL 2	638,073	15%	580	908,987	0.25	10.10	1.16	0.82	2.38	597	596
REGIONAL 3	821,631	19%	853	1,038,179	2.40	14.80	1.04	0.86	5.18	879	878
REGIONAL 4	230,591	5%	227	984,427	0.17	2.80	0.20	0.20	1.72	232	232
REGIONAL 5	162,944	4%	174	1,067,852	0.00	0.46	0.04	0.01	0.01	174	174
**Outpatient**											
REGIONAL 1	2,124,663	58%	650	305,931	0	20.10	0.00	3.15	13.4	688	688
REGIONAL 2	548,237	15%	163	297,317	0	6.07	0.00	0.82	1.03	171	171
REGIONAL 3	657,722	18%	185	281,274	0	10.30	0.01	0.86	4.01	200	200
REGIONAL 4	192,366	5%	55	285,913	0	1.81	0.01	0.20	0.56	58	58
REGIONAL 5	123,663	3%	31.4	253,916	0	0.44	0.00	0.01	0.00	32	32
**Inpatient**											
REGIONAL 1	290,282	47%	1,440	4,960,693	0.12	19.50	5.62	0	4.42	1470	1470
REGIONAL 2	89,836	14%	418	4,652,923	0.25	4.06	1.16	0	1.35	426	425
REGIONAL 3	163,909	26%	668	4,075,432	2.40	4.46	1.03	0	1.17	679	678
REGIONAL 4	38,225	6%	172	4,499,673	0.17	0.99	0.20	0	1.16	175	175
REGIONAL 5	39,281	6%	142	3,614,979	0	0.02	0.04	0	0.01	142	142

Notes:

1. Top-up payments with INA-CBG tariff on Special Casemix Main Groups (CMG), which are: (a) special procedure (SP); (b) special drugs (SD); (c) special investigation (SI); (d) special prosthesis (RR); e.) subacute cases (SA); and f) chronic cases (unavailable)

2. Admission for inpatient care, and visits for outpatient

3. The INA-CBG tariff is grouped into five different regions as follows:

Regional 1: Daerah Istimewa Yogyakarta, Jawa Barat, Jawa Tengah, Jawa Timur, and DKI Jakarta;

Regional 2: NTB, Bengkulu, Sulawesi Tengah, Kalimantan Barat, Lampung, Banten, Sumatera Barat, Bali, and Sumatera Utara;

Regional 3: Sulawesi Tenggara, Jambi, Sulawesi Barat, and Gorontalo;

Regional 4: Kalimantan Selatan, Riau, Kalimantan Tengah, Kalimantan Timur, Kalimantan

Utara, Kepulauan Riau, and Sumatera Selatan;

Regional 5: Sulawesi Selatan, Papua Barat, Papua Selatan, Papua Tengah, Papua Pegunungan, Papua, Papua Barat Daya, Aceh, Kepulauan Bangka Belitung, Sulawesi Utara, Nusa Tenggara Timur, Maluku, and Maluku Utara

## Discussion

We have provided a comprehensive overview of the BPJS-K sample data, highlighting their importance for understanding healthcare utilisation patterns in Indonesia. The descriptive analysis confirms the representativeness of the BPJS-K sample data for both BPJS-K members and Indonesian population. Regional disparities in healthcare utilisation within the national health insurance scheme, especially between the West and East regions, underscores the potential for further examining the sample data to inform targeted interventions.

We have shown that studies using the BPJS-K sample data demonstrate the advantages of administrative records, such as comprehensive documentation and absence of recall bias [12]. BPJS-K sample data has been used to develop risk-prediction models (e.g., pre-eclampsia) [10], and to investigate diagnostic patterns across population groups and geographies. The rich information about healthcare use has also been exploited to examine health services utilisation [12, 14, 15, 17, 18], users’ demographics, service use patterns, referral systems e.g., for dengue fever surveillance system [11], referral pathway compliance [21], and over-reimbursement [16]. There is potential for broader research as exemplified by studies realised in other countries where similar health administrative data is accessible [9, 25–27]. For example, in Asia and the Pacific, Taiwan has highly accessible National Health Insurance reimbursement data and a well-established process for data sharing [27, 28], resulting in about 4,000 studies using this data being identified (1996–2017), primarily on diabetes, stroke, and dementia (top three) but also various medical conditions and interventions [29].

The longitudinal nature of the BPJS-K sample data supports the evaluation of health system interventions such as the introduction of performance-based capitation payments [20]. It enables long-term tracking of individuals’ diagnoses, service use, and responses to health system or environmental changes, potentially up to their lifetime. Similar longitudinal features are found in Korean National Health Information Database [30] and the Taiwan National Health Insurance Research Database [31]. BPJS-K centralised structure also allows continues individual-level data, unlike fragmented data from multiple health insurers in Japan, such as in the JMDC Inc. database [32].

The BPJS-K sample data can be linked to district-level and provincial-level characteristics, enabling analysis across regions, providing a valuable source of information that utilises spatial and temporal heterogeneity in Indonesia. Secure data linkage procedures that comply with data protection regulations could further enhance the utilisation of BPJS-K sample data by connecting to individual-level socioeconomic data. Examples of such secure linkage systems are Belgium’s HISlink project [33] and the integration of survey and administrative National Health Service data in England [34, 35].

The BPJS-K sample data is also available without incurring any fee, which lessens the barriers. This is a key advantage compared to datasets like the US Center for Medicare and Medicaid Services (CMS) data [36], which often require payment.

Besides those benefits and potential uses, some limitations exist as occurred in most administrative data. It lacks details on socioeconomic indicators (e.g. education, income, and employment) compared to household surveys like the Indonesian Family Life Survey (IFLS) [37]. It is missing individual health status, but disability could be inferred from secondary diagnosis in referral care visits. It lacks out-of-pocket payment, information to assess the quality of care (e.g. patient safety and satisfaction) [20], and essential medicines availability [38]. In comparison, the South Korea National Health Insurance database provides more granularity, including income deciles, health behaviours and bio-clinical records, prescriptions and provider-level details on human resources and equipment [39].

BPJS Kesehatan could enhance the use of sample data by providing anonymous provider IDs to capture variations across specific healthcare providers [20] and continuity of care as in the French National Healthcare Insurance database [40]. Improving data quality control and the diagnosis recording system could address challenges faced by coders [41], reducing upcoding issues, which leads to the bill sent to BPJS-K being higher than the service provided by healthcare providers [9, 42], and ensuring high quality diagnosis data.

Countries aiming to provide similar data should prioritise robust technical infrastructure, high data quality, research applicability, and strong political commitment [25].

### Suggestions for data users

Users should note, despite rich healthcare utilisation data the BPJS-K sample data reflects registered member only. Underrepresenting undocumented individuals and unreachable Indigenous communities, due to the requirement of having a national ID and/or a family card. The data underrepresents older adults, and informal-sector workers [43] and lower-middle-class [1] may be excluded, likely due to affordability and eligibility barriers. This may affect generalisability, particularly in regions with low BPJS coverage. The BPJS-K sample data also excludes healthcare utilisation outside the insurance scheme and out-of-pocket payments by BPJS-K members. Triangulating findings with national household surveys like the Indonesian Family Life Survey (IFLS) and the National Socioeconomic Survey (SUSENAS) can help assess discrepancies between administrative records and self-reported healthcare utilisation.

Variables like registration class and segmentation can indicate socioeconomic status and can be used to explore utilisation inequalities to understand additional barriers to access but should be interpreted cautiously. The new standardised inpatient wards (PR No.59/2024) may reduce the granularity of these variables for future analysis. Researchers may think of incorporating alternative socioeconomic indicators by linking regional socioeconomic data (income or employment) or utilising geospatial variables like urban-rural classifications.

Diagnosis recording is likely improving over time but remains unassessed. Carefully handling the data is essential when using diagnostic information. The diagnostic codes in referral healthcare are included only for admissions, and cover primary and secondary diagnosis, with the INA-CBG case-mix code tied only to the primary diagnoses for claim purposes. For a robust analysis, primary and secondary diagnosis codes should be used in combination [44].

### Limitations of the study

This study does not include longitudinal data analysis nor evaluate the quality of routine data recording, which is the source of the BPJS-K sample data. This study focuses on promoting the use of the BPJS-K sample data for research highlighting how the dataset holds value for policy planning and evaluation which can support Indonesia’s central and local governments, as well as non-governmental organisations.

## Conclusion

The BPJS-K sample data, along with its descriptive statistics and potential and limitations discussed suggest scope for policy analysis. The dataset overall represents the national population and covers all districts in Indonesia, although some provinces are over- and under-represented. Our study highlights variations in healthcare utilisation and underscore the data’s considerable potential for health and health systems research, which remains underexploited. We discussed the potential of using more recent data, a wider set of variables, linking district and province-level information, and exploiting the longitudinal dimension. Administrative data from national health insurance schemes like BPJS-K should be further exploited to inform health services and health system frameworks and support policy development. However, improvements in data quality and accessibility, including expanded socioeconomic data and more precise diagnostic recording, are recommended to optimise its research usefulness.

## Supplementary Information

Below is the link to the electronic supplementary material.

Supplementary Material 1

## Data Availability

The BPJS-K sample data is accessible upon request to BPJS Kesehatan through their data portal, which access is restricted to internet protocol (IP) addresses within Indonesia. Users must register on the portal, and non-Indonesian users are required to collaborate with an Indonesian researcher, as registration requires a BPJS-K members ID number. Once registered, individuals complete a data request form and sign an integrity pact. A download link will be automatically sent to registered email address. BPJS-K requests that data users do not distribute the sample data to others without written agreement from BPJS-K and to submit the final research outputs through the portal.

## Abbreviations

BPJS-K: Badan Penyelenggara Jaminan Sosial Kesehatan or National Health Insurance Agency
PHC: Primary healthcare
RHC: Referral healthcare
INA-CBG: Indonesian Case-Based Groups
PR: Presidential Regulation
IDR: Indonesian Rupiah
USD: United States Dollars
HMR: Health Minister Regulation
ICD-10: International Statistical Classification of Diseases and Related Health Problems 10^th^ Revision
IFLS: Indonesian Family Life Survey
SUSENAS: Survey Sosial Ekonomi Nasional or National Socioeconomic survey
RISKESDAS: Riset Kesehatan Dasar or Basic health research
