# Supplementary material for: Indonesian National Health Insurance scheme longitudinal sample data 2015–2020: overview and potential uses for health policy analysis
Source: BMC Health Serv Res. 2025 Dec 12;25:1593. doi: 10.1186/s12913-025-13756-9 (PMC12699834; doi:10.1186/s12913-025-13756-9)
Supplement: Supplementary file 1 — Supplementary Material 1 [file 12913_2025_13756_MOESM1_ESM.docx]

**Appendix**

**Table of Contents**

[A1. Systematic search of studies using the BPJS-K sample data 2](#_Toc194488785)

[A2. Sampling method description 5](#_Toc194488786)

[A3. Results and additional information 7](#_Toc194488787)

[Tables 7](#_Toc194488788)

[Figures 23](#_Toc194488789)

**List of tables**

[Table 1 Number of studies retrieved 2](#_Toc193896433)

[Table 2 Studies using BPJS-K sample data 3](#_Toc193896434)

[Table 3 Studies using BPJS-K claims data 4](#_Toc193896435)

[Table 4 Individuals in sample data 7](#_Toc193896436)

[Table 5 Data dictionary for memberships dataset 7](#_Toc193896437)

[Table 6 Membership status for the 2017-2018 and 2019-2020 period 8](#_Toc193896438)

[Table 7 Type and ownership of primary healthcare provider of registration 9](#_Toc193896439)

[Table 8 Chi-square statistics of sample and census data distribution 10](#_Toc193896440)

[Table 9 Members ever accessed health facilities through the BPJS-K insurance program, 2015-2020 11](#_Toc193896441)

[Table 10 Variables in primary healthcare visit datasets 11](#_Toc193896442)

[Table 11 Primary healthcare visits for services under capitation by year 13](#_Toc193896443)

[Table 12 Number of primary healthcare visits for services under capitation by discharge status, 2015-2020 14](#_Toc193896444)

[Table 13 Primary healthcare visits under non-capitation services, by year 15](#_Toc193896445)

[Table 14 Primary healthcare visits under non-capitation services by type of treatment 16](#_Toc193896446)

[Table 15 Primary healthcare visits within non-capitation: Total treatment cost (in Million Rupiah) 17](#_Toc193896447)

[Table 16 Referral health care providers visit dataset 18](#_Toc193896448)

[Table 17 Referral healthcare secondary diagnostic dataset 20](#_Toc193896449)

[Table 18 Referral healthcare visits by year 21](#_Toc193896450)

[Table 19 Referral healthcare: Most common primary diagnosis, 2015-2020 22](#_Toc193896451)

[Table 20 Referral healthcare: Most common secondary diagnoses, 2005-2020 23](#_Toc193896452)

**List of Figures**

[Figure A2. 1 Ratio of individuals in the data to total BPJS-K members, by Province 2019-2020 6](#_Toc193896712)

[Figure 1 Revision to the Presidential Regulation on National Health Insurance 2013-2024 23](#_Toc193896691)

[Figure 2 Rate of sample-weighted individuals who visited PHC in 2015-2020 by 10,000 BPJS-K members in quartile groups 24](#_Toc193896692)

[Figure 3 Number of diagnoses based on the number of primary healthcare visits per diagnosis for services under capitation, 2015-2020 26](#_Toc193896693)

[Figure 4 Most common diagnosis of primary healthcare visits for services under capitation, 2015 – 2020 27](#_Toc193896694)

[Figure 5 Referral healthcare: Number of primary diagnosis based on the number of visits per diagnosis, 2015-2020 28](#_Toc193896695)

# A1. Systematic search of studies using the BPJS-K sample data

**Table 1 Number of studies retrieved**

| **Keywords** | **Studies retrieved from the search through:** | | | | | | |
| --- | --- | --- | --- | --- | --- | --- | --- |
|  | **Pubmed** | **Google Scholar** | **CORE** | **SSRN** | **MPRA** | **Science Direct** | **Taylor and Francis*** |
| **Additional category** |  | **Article** | **Research** |  |  | **Research** | **Article** |
| BPJS AND sample data | 5 | 6 | 10 | 1 | 1 | 89 | 0 |
| Indonesian AND National AND Health AND Insurance AND (INHI) AND “sample data” | 1 | 160 | 52 | 0 | 0 | 0 | 76 |
| Jaminan AND Kesehatan AND Nasional | 27 | 691 | 111 | 1 | 0 | 27 | 54 |
| JKN | 393 | 873 | 77 | 2 | 0 | 125 | 100 |
| Badan AND Penjamin AND Jaminan AND Kesehatan | 0 | 35 | 19 | 0 | 0 | 0 | 0 |
| “National Health Insurance” AND Indonesia | 125 | 603 | 12 | 3 | 1 | 187 | 100 |
| universal AND health AND coverage AND Indonesia | 108 | 1330 | 71 | 5 | 21 | 819 | 100 |
| “Social security agency” AND health AND Indonesia | 23 | 73 | 169 | 1 | 0 | 25 | 0 |

Note:

Google Scholar, Pubmed and CORE showed results not only in English but also in Bahasa Indonesia, whilst the rest are strictly articles in English even when the keywords are in Bahasa Indonesia

*Taylor and Francis show only the top 100 articles

**Table 2 Studies using BPJS-K sample data**

| **No** | **Title** | **Authors** | **Publication Year** | **Journal** | **BPJS-K sample data** | **Level of analysis** |
| --- | --- | --- | --- | --- | --- | --- |
| 1 | Artificial intelligence-assisted prediction of preeclampsia: Development and external validation of a nationwide health insurance dataset of the BPJS Kesehatan in Indonesia(1) | H. Sufriyana, Y. W. Wu, and E. C. Y. Su | 2020 | BioMedicine | 2015-2016 | Individual and visits |
| 2 | Improving dengue surveillance system with administrative claim data in Indonesia: Opportunities and Challenges (2) | A. Husnayain, A. Fuad, I. S. Laksono, and E. C. Y. Su | 2020 | Studies in health technology and informatics | 2015-2016 | Individual, In-patient cases of Dengue fever |
| 3 | Multimorbidity patterns of chronic diseases among Indonesians: Insights from Indonesian national health insurance (INHI) sample data (3) | A. Husnayain, N. Ekadinata, D. Sulistiawan, and E. C. Y. Su | 2020 | International Journal of Environmental Research and Public Health | 2015-2016 | Individual, visits |
| 4 | The Regional And Referral Compliance of Online Healthcare Systems By Indonesia National Health Insurance Agency And Health-Seeking Behavior In Indonesia (4) | P. W. Handayani et al. | 2021 | Heliyon | 2015-2016, with additional analysis using survey data | provincial |
| 5 | A population-based study on coverage and healthcare processes for cancer during the implementation of national healthcare insurance in Indonesia (5) | J. Schaefers, S. Wenang, A. Afdal, A. G. Mukti, S. Sundari, and J. Haier | 2022 | Lancet Regional Health Southeast Asia | 2018, combined with other statistical published data from Statistics Indonesia | Provincial |
| 6 | Effects of performance-based capitation payment on the use of public primary health care services in Indonesia (6) | N. P. Sambodo, I. Bonfrer, R. Sparrow, M. Pradhan, and E. van Doorslaer | 2023 | Social Science and Medicine | 2015-2016 | District |
| 7 | Temperature and non-communicable diseases: Evidence from Indonesia's primary health care system (7) | M. Fritz | 2022 | Health Economics | 2015-2016, combined with SA-OBS dataset weather data | District |
| 8 | The difference in Diabetes Mellitus’s primary healthcare utility after Performance-Based Capitation payment in the city of Cimahi (Analysis using BPJS sample data 2016-2020) (8) | E. I. Gultom, I. Afriandi, and S. Gondodiputro | 2023 | Jurnal Kebijakan Kesehatan Indonesia | 2015-20 | Individual, visits |
| 9 | JKN Cost Analysis Analisis for hypertension patients of primary healthcare providers in West Java for the year 2015-2016 (9) | E. Dewi, Y. Sofiatin, E. Setiawati, K. Wahyudi, and I. Afriandi | 2021 | Jurnal Kebijakan Kesehatan Indonesia | 2015-2016 | Individual, visits |
| 10 | The hemodialysis service utility of National Health Insurance members (Analysis of BPJS data sample year 2015-2016) (10) | I. Fitrilia | 2021 | Master Thesis University of Gadjah Mada | 2015-2016 | Individual, visits |
| 11 | The pattern of service utility of hypertension patient who is National Health Insurance member in referral healthcare providers in West Java province year 2015-2016 (11) | W. Fitrian, Y. Sofiatin, and I. Afriandi | 2021 | Jurnal Kebijakan Kesehatan Indonesia | 2015-2016 | Individual, visits |
| 12 | Utilization patterns of healthcare facility and estimated expenditure of PLHIV care under the Indonesian National Health Insurance Scheme in 2018(12) | Setiawan, Ery  Nurjannah, Nurjannah  Komaryani, Kalsum  Nugraha, Ryan Rachmad  Thabrany, Hasbullah  Purwaningrum, Farah  Sarnianto, Prih | 2022 | BMC Health Services Research | 2018 | Individual, visits |

**Table 3 Studies using BPJS-K claims data**

| **No** | **Title** | **Authors** | **Publication Year** | **Journal** | **Data year** | **Level of analysis** |
| --- | --- | --- | --- | --- | --- | --- |
| 1 | National Health Insurance Databases in Indonesia, Vietnam and the Philippines (13) | Ng, Junice Yi Siu  Ramadani, Royasia Viki  Hendrawan, Donni  Duc, Duong Tuan  Kiet, Pham Huy Tuan | 2019 | PharmacoEconomics - Open | 2014 and 2017 |  |
| 2 | Income Disparity and Healthcare Utilization: Lessons from Indonesia’s National Health Insurance Claim Data (14) | Nugroho, Sony Tito  Ahsan, Abdillah  Kusuma, Dian  Adani, Nadhila  Irawaty, Dian Kristiani  Amalia, Nadira  Hati, Sri Rahayu Hijrah | 2023 | Asian Pacific Journal of Cancer Prevention | 2015 and 2016 | Individuals, visits |
| 3 | Evaluation of health care quality among insured patients in Indonesian mother & child hospital: A secondary data analysis(15) | Rachmawaty, Rini  Sinrang, Andi Wardihan  Wahyudin, Elly  Bukhari, Agussalim | 2021 | Gaceta Sanitaria | e-claim database from January 1 to December 31, 2019, and from January 1 to June 30, 2020. | Individuals, visits |
| 4 | Effects of the COVID-19 pandemic on cardiovascular disease financing in Indonesia (JKN claims data analysis 2019–2020) | Nugraheni, Wahyu Pudji  Retnaningsih, Ekowati  Mubasyiroh, Rofingatul  Rachmawati, Tety | 2023 | Frontiers in Public Health | 2019, 2020 | Individuals, visits |
| 5 | Does geographic spending variation exacerbate healthcare benefit inequality? A benefit incidence analysis for Indonesia (16) | Sambodo, Novat Pugo  Van Doorslaer, Eddy  Pradhan, Menno  Sparrow, Robert | 2021 | Health Policy and Planning | 2015, 2016, and 2017 combined with SUSENAS in the same years | District |
| 6 | The sensitivity of hospital coding to prices: evidence from Indonesia. (17) | Chalkley, Martin  Hidayat, Budi  Ramadani, Royasia Viki  Aragón, María José | 2022 | International journal of health economics and management | 2015 and 2017 | Individuals, visit |

# A2. Sampling method description

The BPJS-K sample data represents all BPJS-K members in Indonesia, each uniquely registered with one BPJS-K contracted primary healthcare providerv(PHC). Upon registration, BPJS-K members can select their PHC provided it has sufficient capacity, which typically based on proximity to their residence to ensure accessibility and fostering continuity of care. . Members may also swithc to other PHCs within three months after registering at a PHC.

During the 2015-2020, 60% of members registered at PUSKESMAS (government-run health centres), and the remainder were private, army and police primary healthcare clinics (Appendix Table 7).

The sampling frame consists of all BPJS-registered households because registration requires entire household members listed in family card to enroll together.

The sample is stratified by PHC and three types of household: one for household with members who have never had a PHC visit recorded, and two groups for households with members who have ever had a PHC visit recorded and who have ever had PHC and RHC visit recorded.

The 2015-2016 data includes a sample drawn from members registered up to 31 December 2016, covering 22,024 PHC and 66,072 (22,024 X 3) household groups constituting the sampling frame. Ten households were randomly drawn per stratum. If the stratum had fewer than 10 households, all households were included, resulting in 586,969 households (89% of the total 660,720 households if all strata had at least 10 households).

To reflect for expanded coverage and household composition changes, the BPJS-K sample data is boosted with new PHC facilities and new registered household members after the initial 2015-2016 period. In all PHCs, including new ones, one newly registered household (in addition to the 10 initially included in the 2015-2016 period) is drawn per stratum. In all periods, all new household members are included in the sample data.

The dataset provides the household sample weight, which is the inverse of the probability of a household being chosen out of all registered households in a stratum (household group within a PHC), and individual sample weight. Initially, individual weight were based on household weight and household size in 2015-2016 . Later, they were adjusted by multiplying household weight with a constant number that reflect sample representativeness to the total population by sex, age, and membership segmentation.

Figure A2.1 below also shows the sampling geographic distribution, with some provinces experiencing under- and over-representation.

**Figure A2. 1 Ratio of individuals in the data to total BPJS-K members by Province 2019-2020**


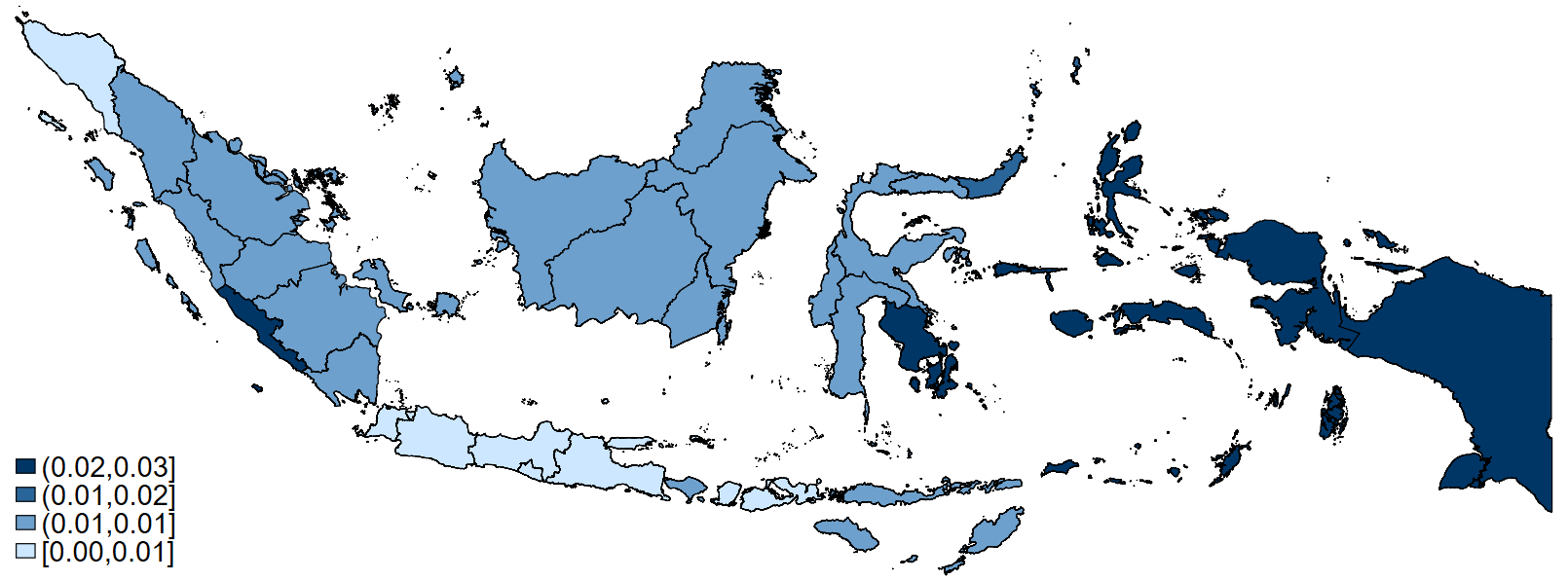


Source: BPJS-K Sample data, author calculation. The BPJS-K registered members by province are from the National Insurance Board ([DJSN](https://kesehatan.djsn.go.id/kesehatan/cakupan/)) website.

# A3. Descriptive tables and figures

## Tables

**Table 4 Individuals in sample data**

| **Number of Individuals sampled** | **%** | **Pattern** | | |
| --- | --- | --- | --- | --- |
|  |  | **2015-2016** | **2017-2018** | **2019-2020** |
| 1,697,452 | 77.12 | Included in the sample | Included in the sample | Included in the sample |
| 274,292 | 12.46 |  | Included in the sample | Included in the sample |
| 229,216 | 10.41 |  |  | Included in the sample |

**Table 5 Data dictionary for memberships dataset**

| **Variables** | **Variables name in Bahasa Indonesia** | **Variables name in English** |
| --- | --- | --- |
| PSTV01 | Nomor peserta | Person id |
| PSTV02 | Nomor keluarga | Household id |
| PSTV03 | Tanggal lahir peserta | Date of birth |
| PSTV04 | Hubungan Keluarga | Household relationship |
| PSTV05 | Jenis Kelamin | Sex |
| PSTV06 | Status perkawinan | Marital status |
| PSTV07 | Kelas rawat | Inpatient ward class |
| PSTV08 | Segmentasi peserta | Member’s segmentation |
| PSTV09 | Provinsi Tempat Tinggal Peserta | The province where the person lives |
| PSTV10 | Kabupaten/Kota Tempat Tinggal Peserta | District the person lives |
| PSTV11 | Kepemilikan faskes | Ownership of health facility (i.e. public or private) |
| PSTV12 | Jenis faskes | Type of health facility (i.e. Puskesmas, clinic, general practice) |
| PSTV13 | Provinsi Fasilitas Kesehatan Peserta Terdaftar | Province registered |
| PSTV14 | Kabupaten/Kota Fasilitas Kesehatan Peserta Terdaftar | District registered |
| PSTV15 | Bobot | Weight |
| PSTV16 | Tahun Sampel | Sampling year |
| PSTV17 | Status Kepesertaan | Membership status |
| PSTV18 | Tahun Meninggal | Year of death |

Note: These variables are consistent in different data tranches from 2015 to 2020. They were updated in each period. The inactive status of the individual is recorded in the membership dataset at a given point in time and does not always translate directly to no visits to PHC throughout that period. ***Table 6*** shows this dynamic and reporting the changes of membership status from 2017-2018 to 2019-2020 period.

**Table 6 Membership status for the 2017-2018 and 2019-2020 period**

| **Membership status** | **Frequency** | **Percentage** |
| --- | --- | --- |
| Active in both periods of 2017-2018 and 2019-2020 | 1,263,414 | 57·403% |
| Active in 2017-2018 to Inactive in 2019-2020 | 248,447 | 11·288% |
| Inactive in 2017-2018 to Active in 2019-2020 | 88,275 | 4·011% |
| Inactive in both periods | 326,918 | 14·853% |
| Active in 2017-2018 to death in 2019-2020 | 15,428 | 0·701% |
| Inactive in 2017-2018 to death in 2019-2020 | 2,865 | 0·130% |
| Death in 2017-2018 to inactive in 2019-2020 | 126 | 0·006% |
| Death in 2017-2018 to active in 2019-2020 | 55 | 0·002% |
| Death in both periods | 26,216 | 1·191% |

Source: BPJS sample data 2015-2016, 2017-2018, and 2019-2020, author calculation

Note: The membership dataset is updated at the period of the sample. The period of 2019-2020 means updated membership information snapshot for people during the year of 2019 and 2020. There is no information on the exact time when the snapshot is made.

**Table 7 Type and ownership of primary healthcare provider of registration**

| **Providers ownership** | **Type of primary providers** | **2015-2016** | **%** | **2017-2018** | **%** | **2019-2020** | **%** |
| --- | --- | --- | --- | --- | --- | --- | --- |
| Central government | PRIMARY CLINIC | 11 | 0.00% | 208 | 0.01% | 387 | 0.02% |
| Provincial Government | PUSKESMAS | 6,633 | 0.39% | 8,861 | 0.45% | 9,864 | 0.45% |
|  | PRIMARY CLINIC | 544 | 0.03% | 570 | 0.03% | 729 | 0.03% |
|  | GENERAL PRACTICIONER | 7 | 0.00% |  | 0.00% |  | 0.00% |
| District Government | PUSKESMAS | 921,030 | 54.26% | 1,144,438 | 58.04% | 1,303,125 | 59.21% |
|  | PRIMARY CLINIC | 1,942 | 0.11% | 2,090 | 0.11% | 2,204 | 0.10% |
|  | GENERAL PRACTICIONER | 169 | 0.01% | 9 | 0.00% | 9 | 0.00% |
|  | MISSING |  | 0.00% | 2 | 0.00% | 2 | 0.00% |
| POLRI - Police | PRIMARY CLINIC | 43,498 | 2.56% | 42,503 | 2.16% | 43,962 | 2.00% |
|  | MISSING |  | 0.00% | 1 | 0.00% | 1 | 0.00% |
| TNI AD -Army | PRIMARY CLINIC | 38,104 | 2.24% | 36,423 | 1.85% | 37,762 | 1.72% |
| TNI AL - Marine | PRIMARY CLINIC | 10,239 | 0.60% | 9,133 | 0.46% | 9,358 | 0.43% |
| TNI AU - Air force | PRIMARY CLINIC | 6,236 | 0.37% | 6,265 | 0.32% | 6,469 | 0.29% |
| BUMN – Government-owned company | PRIMARY CLINIC | 15,248 | 0.90% | 18,602 | 0.94% | 23,512 | 1.07% |
|  | GENERAL PRACTICIONER | 1,473 | 0.09% | 1,063 | 0.05% | 889 | 0.04% |
| Private | PUSKESMAS | 93 | 0.01% | 23 | 0.00% | 22 | 0.00% |
|  | PRIMARY CLINIC | 277,494 | 16.35% | 343,346 | 17.41% | 418,291 | 19.00% |
|  | GENERAL PRACTICIONER | 373,342 | 21.99% | 343,185 | 17.41% | 342,522 | 15.56% |
|  | MISSING |  | 0.00% | 5 | 0.00% | 2 | 0.00% |
| MISSING | PUSKESMAS | 179 | 0.01% | 35 | 0.00% | 34 | 0.00% |
|  | PRIMARY CLINIC | 51 | 0.00% | 19 | 0.00% | 7 | 0.00% |
|  | GENERAL PRACTICIONER | 1,159 | 0.07% | 389 | 0.02% | 383 | 0.02% |
|  | MISSING |  | 0.00% | 14,574 | 0.74% | 1,426 | 0.06% |
| **Total** |  | **1,697,452** | **100%** | **1,971,744** | **100%** | **2,200,960** | **100%** |

**Table 8 Chi-square statistics of sample and census data distribution**

|  | **Obs** | **Chi-Square stat** | **Degree of freedom** | **P-value** |
| --- | --- | --- | --- | --- |
| Male | 16 | 0.799 | 15 | 0.99 |
| Female | 16 | 1.034 | 15 | 0.99 |

Note:

Chi-square calculated as:

$${Chi}^{2}= \frac{\sum{({BPJS}_{i}- {Census}_{i})}^{2}}{{Census}_{i}}$$

Where i is a different age category and BPJS and Census refer to the percentage of individuals in an age category out of the total individuals in BPJS sample data and census data. The chi-square statistics and corresponding p-values indicate no statistically significant differences between the BPJS-K sample data and the census data for both male and female distributions across age categories. This suggests that the BPJS-K sample is representative of the Indonesian population in terms of age and sex distribution.

**Table 9 Members ever accessed health facilities through the BPJS-K insurance program, 2015-2020**

| **Sample year** | **Never-accessed member** | **Cumulative never-accessed member** | **Cumulative total number of observations** | **% Never-accessed over total observation** |
| --- | --- | --- | --- | --- |
| (1) | (2) | (3) | (4) | (3)/(4)x100% |
| 2015-2016 | 622,835 | 622,835 | 1,697,452 | 37% |
| 2017 | +48,169 | 671,004 | 1,832,418 | 37% |
| 2018 | +58,261 | 729,265 | 1,971,744 | 37% |
| 2019 | +56,594 | 785,859 | 2,093,156 | 38% |
| 2020 | +62,477 | 848,336 | 2,200,960 | 39% |

Note: We stacked the membership data in all periods together and merged them with visits data, in which we identified individuals who did not have any visits with healthcare providers across the panel years of 2015 to 2020.

**Table 10 Variables in primary healthcare visit datasets**

| **Variables Capitation** | **Variables Non-Capitation** | **Variable labels in Bahasa Indonesia** | **Variable labels in English** |
| --- | --- | --- | --- |
| PSTV01 | PSTV01 | Nomer peserta | Pseudo-anonymised member ID |
| PSTV02 | PSTV02 | ID keluarga | Household ID |
| PSTV15 | PSTV15 | Bobot | Sample weight |
| FKP02 | PNK02 | ID kunjungan | Visit ID |
| FKP03 | PNK03 | Datang kunjungan FKTP | Admission date |
|  | PNK04 | Tanggal tindakan | Treatment date |
| FKP04 | PNK05 | Pulang kunjungan FKTP | Discharge date |
| FKP05 | PNK06 | Provinsi FKTP | Province of PHC |
| FKP06 | PNK07 | Kabupaten/Kota FKTP | District of PHC |
| FKP07 | PNK08 | Kepemilikan FKTP | Ownership of PHC |
| FKP08 | PNK09 | Jenis FKTP | Type of PHC (i.e. Puskesmas, clinic, lab) |
| FKP09 | PNK10 | Tipe FKTP | Type of PHC (i.e. inpatient, non-inpatient, lab) |
| FKP10 | PNK11 | Tingkat Pelayanan FKTP | Degree of service (i.e. primary outpatient, primary inpatient, promotive) |
| FKP11 |  | Jenis Poli FKTP | Type of outpatient clinic in the PHC |
| FKP12 | PNK12 | Segmen Peserta saat akses layanan FKTP | Membership segmentation |
| FKP13 |  | Status Pulang peserta | Discharge status |
| FKP14 | PNK13 | Nama diagnosis | Diagnosis name |
|  | PNK13A | Kode diagnosis berdasarkan ICD-10 (3 digit) | Diagnosis code based on ICD-10 (3 digits) |
| FKP14A |  | Kode dan nama diagnosis | Code and name of diagnosis |
| FKP15 | PNK14 | Kode dan nama diagnosis ICD-10 (3-5 digit) | Code and name of diagnosis ICD-10 (3-5 digits) |
| FKP15A | PNK15 | Nama diagnosis dari kode diagnosis | Name of diagnosis |
|  | PNK16 | Nama Tindakan | Name of treatment |
|  | PNK17 | Biaya tagih | Treatment cost claimed |
|  | PNK18 | Biaya verifikasi | Treatment cost verified |
| FKP16 |  | Provinsi faskes tujuan rujukan | Province of referral healthcare provider |
| FKP17 |  | Kabupaten/Kota faskes tujuan rujukan | District of referral healthcare provider |
| FKP18 |  | Kepemilikan faskes tujuan rujukan | Ownership of referral healthcare provider |
| FKP19 |  | Jenis faskes tujuan rujukan | Type of referral healthcare provider |
| FKP20 |  | Tipe faskes tujuan rujukan | Type of referral healthcare provider |
| FKP21 |  | Poli faskes tujuan rujukan | Type of outpatient clinic of referral healthcare provider |
| FKP22 |  | Jenis Kunjungan FKTP | Type of PHC visit, healthy or sick visits |

Note: Some variables are included in primary healthcare visits for capitation services but not in non-capitation services, such as information on referrals. Similarly, information on the treatment and treatment cost are in the non-capitation visits dataset but not in the capitation visit dataset. All variables are consistent across periods.

**Table 11 Primary healthcare visits for services under capitation by year**

| **Visit year** | **Frequency visits** | **Frequency individuals** | **Weighted frequency visits** | **Weighted frequency individuals** |
| --- | --- | --- | --- | --- |
| 2015 | 687,957 | 251,834 | 54,931,106 | 19,741,566 |
| 2016 | 1,045,802 | 356,137 | 80,150,058 | 27,052,551 |
| 2017 | 1,978,544 | 563,408 | 193,315,236 | 57,222,340 |
| 2018 | 2,339,282 | 628,856 | 241,831,770 | 65,350,138 |
| 2019 | 2,726,234 | 695,145 | 287,870,813 | 73,475,924 |
| 2020 | 2,344,889 | 615,759 | 247,931,517 | 63,248,405 |

**Table 12 Number of primary healthcare visits for services under capitation by discharge status, 2015-2020**

| **Discharge status** | **2015** | **2016** | **2017** | **2018** | **2019** | **2020** |
| --- | --- | --- | --- | --- | --- | --- |
| Outpatient | 596,423 | 924,412 | 1,263,579 | 1,315,951 | 1,503,592 | 1,313,270 |
|  | 87% | 88% | 64% | 56% | 55% | 56% |
| Referred | 64,327 | 97,118 | 170,197 | 231,748 | 267,644 | 206,334 |
|  | 9% | 9% | 9% | 10% | 10% | 9% |
| Recovered | 26,159 | 23,022 | 25,312 | 37,957 | 31,850 | 18,502 |
|  | 4% | 2% | 1% | 2% | 1% | 1% |
| Referred internally | 665 | 834 | 1,120 | 184 | 618 |  |
|  | 0% | 0% | 0% | 0% | 0% | 0% |
| Voluntary discharged | 172 | 207 | 170 | 152 | 276 | 177 |
|  | 0% | 0% | 0% | 0% | 0% | 0% |
| Others | 184 | 181 | 420 | 678 | 1,948 | 555 |
|  | 0% | 0% | 0% | 0% | 0% | 0% |
| Dead | 27 | 26 | 23 | 29 | 121 | 83 |
|  | 0% | 0% | 0% | 0% | 0% | 0% |
| missing |  | 2 |  |  |  |  |
|  | 0% | 0% | 0% | 0% | 0% | 0% |
| Healthy visit |  |  | 517,723 | 752,583 | 920,185 | 805,968 |
|  | 0% | 0% | 26% | 32% | 34% | 34% |
| Total Visit | 687,957 | 1,045,802 | 1,978,544 | 2,339,282 | 2,726,234 | 2,344,889 |
|  | 100% | 100% | 100% | 100% | 100% | 100% |

**Table 13 Primary healthcare visits under non-capitation services, by year**

| **Visit year** | **Frequency visits** | **Frequency individuals** | **Weighted frequency visits** | **Weighted frequency individuals** |
| --- | --- | --- | --- | --- |
| 2015 | 43,477 | 33,546 | 3,209,712 | 1,339,395 |
| 2016 | 60,987 | 43,210 | 4,396,720 | 1,794,037 |
| 2017 | 58,635 | 50,036 | 4,764,342 | 1,632,022 |
| 2018 | 76,627 | 53,994 | 5,983,057 | 1,889,420 |
| 2019 | 86,968 | 58,373 | 6,684,900 | 2,202,208 |
| 2020 | 79,626 | 54,818 | 5,878,993 | 2,227,537 |

**Table 14 Primary healthcare visits under non-capitation services by type of treatment**

| **Treatment** |  | **2015** | **2016** | **2017** | **2018** | **2019** | **2020** |
| --- | --- | --- | --- | --- | --- | --- | --- |
| Ambulance ^ | Frequency | 820 | 1,093 | 1,952 | 2,202 | 2,641 | 2,320 |
|  | Proportion | 2% | 2% | 3% | 3% | 3% | 3% |
| Inpatient treatment | Frequency | 22,159 | 20,752 | 516 | 40 | 2,116 | 3,013 |
|  | Proportion | 51% | 34% | 1% | 0% | 2% | 4% |
| Outpatient treatment | Frequency | 4 | 23 |  |  |  |  |
|  | Proportion | 0% | 0% | 0% | 0% | 0% | 0% |
| Cholesterol related check | Frequency |  | 546 | 2,935 | 13,581 | 23,027 | 23,295 |
|  | Proportion | 0% | 1% | 5% | 18% | 26% | 29% |
| Blood glucose-related check | Frequency | 1,224 | 9,365 | 14,966 | 13,916 | 9,802 | 9,134 |
|  | Proportion | 3% | 15% | 26% | 18% | 11% | 11% |
| Vaginal birth | Frequency | 8,617 | 10,003 | 8,969 | 8,718 | 8,237 | 9,291 |
|  | Proportion | 20% | 16% | 15% | 11% | 9% | 12% |
| Antenatal care | Frequency | 1,847 | 4,273 | 9,221 | 8,913 | 6,053 | 2,285 |
|  | Proportion | 4% | 7% | 16% | 12% | 7% | 3% |
| Postnatal care | Frequency | 4,119 | 7,085 | 8,614 | 8,099 | 5,069 | 1,310 |
|  | Proportion | 9% | 12% | 15% | 11% | 6% | 2% |
| Pre-referral treatment (maternity & neonatal complication) | Frequency | 297 | 378 | 741 | 672 | 683 | 646 |
|  | Proportion | 1% | 1% | 1% | 1% | 1% | 1% |
| Bleeding after miscarriage | Frequency | 4 | 5 | 2 |  | 2 |  |
|  | Proportion | 0% | 0% | 0% | 0% | 0% | 0% |
| Contraceptive | Frequency | 4,263 | 6,241 | 8,127 | 8,866 | 9,348 | 7,946 |
|  | Proportion | 10% | 10% | 14% | 12% | 11% | 10% |
| Microalbuminuria | Frequency |  | 160 | 675 | 3,249 | 5,590 | 5,738 |
|  | Proportion | 0% | 0% | 1% | 4% | 6% | 7% |
| Blood urea nitrogen test | Frequency |  | 168 | 702 | 3,373 | 5,734 | 5,817 |
|  | Proportion | 0% | 0% | 1% | 4% | 7% | 7% |
| HbA1c | Frequency |  | 428 | 501 | 1,621 | 2,932 | 2,997 |
|  | Proportion | 0% | 1% | 1% | 2% | 3% | 4% |
| Creatinin | Frequency |  | 157 | 714 | 3,377 | 5,734 | 5,834 |
|  | Proportion | 0% | 0% | 1% | 4% | 7% | 7% |
| Triglycerides | Frequency |  | 182 |  |  |  |  |
|  | Proportion | 0% | 0% | 0% | 0% | 0% | 0% |
| Other blood-related treatment/test | Frequency | 4 |  |  |  |  |  |
|  | Proportion | 0% | 0% | 0% | 0% | 0% | 0% |
| Other treatment* | Frequency | 118 | 127 |  |  |  |  |
|  | Proportion | 0% | 0% | 0% | 0% | 0% | 0% |
| Total | Frequency | 43,476 | 60,986 | 58,635 | 76,627 | 86,968 | 79,626 |
|  | Proportion | 100% | 100% | 100% | 100% | 100% | 100% |
| ^ Ambulance services is for transporting BPJS patients between primary healthcare providers, or from primary healthcare providers to referral healthcare providers | | | | | | | |
| *including hecting, nebulizer, catheterisation, open wound treatment, Widal test, Acetowhite test, maternity treatment related to illnesses | | | | | | | |

**Table 15 Primary healthcare visits within non-capitation: Total treatment cost (in Million Rupiah)**

| **Treatment** | **2015** | | **2016** | | **2017** | | **2018** | | **2019** | | **2020** | |
| --- | --- | --- | --- | --- | --- | --- | --- | --- | --- | --- | --- | --- |
|  | **Treatment cost** | **verification cost** | **Treatment cost** | **verification cost** | **Treatment cost** | **verification cost** | **Treatment cost** | **verification cost** | **Treatment cost** | **verification cost** | **Treatment cost** | **verification cost** |
| Ambulance | 269.67 | 269.67 | 379.09 | 379.09 | 564.20 | 564.20 | 653.56 | 653.56 | 802.43 | 802.43 | 751.88 | 751.88 |
| Inpatient treatment | 7,859.6 | 7,859.66 | 8,841.3 | 8,841.37 | 42.84 | 42.84 | 19.05 | 19.05 | 963.72 | 963.72 | 1,419.9 | 1,419.92 |
| Outpatient treatment | 0.18 | 0.18 | 2.63 | 2.63 | 0.00 | 0.00 | 0.00 | 0.00 | 0.00 | 0.00 | 0.00 | 0.00 |
| Blood cholesterol test | 0.00 | 0.00 | 242.76 | 242.76 | 145.37 | 145.37 | 673.17 | 673.17 | 1134.75 | 1134.75 | 1140.07 | 1140.07 |
| Blood glucose test | 39.22 | 39.22 | 340.15 | 340.15 | 276.67 | 276.67 | 247.88 | 247.88 | 171.56 | 171.56 | 162.00 | 162.00 |
| Vaginal birth | 5,345.6 | 5,345.60 | 6,108.8 | 6,108.84 | 6,094.7 | 6,094.75 | 6,150.1 | 6,150.15 | 5,796.3 | 5,796.38 | 6,539.6 | 6,539.65 |
| Antenatal care | 599.35 | 599.35 | 814.32 | 814.32 | 460.68 | 460.68 | 445.53 | 445.53 | 302.43 | 302.43 | 114.20 | 114.20 |
| Postnatal care | 798.32 | 798.32 | 521.59 | 521.59 | 219.10 | 219.10 | 206.38 | 206.38 | 128.50 | 128.50 | 33.43 | 33.43 |
| Pre-referral treatment (maternity & neonatal complication) | 53.85 | 53.85 | 62.80 | 62.80 | 92.63 | 92.63 | 84.00 | 84.00 | 85.38 | 85.38 | 80.63 | 80.63 |
| Bleeding after miscarriage | 3.00 | 3.00 | 3.75 | 3.75 | 1.50 | 1.50 | 0.00 | 0.00 | 1.50 | 1.50 | 0.00 | 0.00 |
| Contraceptive | 180.68 | 180.68 | 207.29 | 207.29 | 234.81 | 234.81 | 263.24 | 263.24 | 298.14 | 298.14 | 285.62 | 285.62 |
| Microalbuminuria | 0.00 | 0.00 | 73.24 | 73.24 | 80.97 | 80.97 | 389.31 | 389.31 | 668.73 | 668.73 | 683.99 | 683.99 |
| Blood urea nitrogen test | 0.00 | 0.00 | 75.08 | 75.08 | 20.99 | 20.99 | 100.90 | 100.90 | 171.21 | 171.21 | 173.13 | 173.13 |
| HbA1c | 0.00 | 0.00 | 106.46 | 106.46 | 80.46 | 80.46 | 261.93 | 261.93 | 472.26 | 472.26 | 482.64 | 482.64 |
| Creatinin | 0.00 | 0.00 | 68.47 | 68.47 | 21.35 | 21.35 | 101.02 | 101.02 | 168.60 | 168.60 | 171.99 | 171.99 |
| Triglycerides | 0.00 | 0.00 | 80.72 | 80.72 | 0.00 | 0.00 | 0.00 | 0.00 | 0.00 | 0.00 | 0.00 | 0.00 |
| Other blood-related test | 2.50 | 2.50 | 0.36 | 0.36 | 0.00 | 0.00 | 0.00 | 0.00 | 0.00 | 0.00 | 0.00 | 0.00 |
| Other treatment* | 35.16 | 35.16 | 36.21 | 36.21 | 0.00 | 0.00 | 0.00 | 0.00 | 0.00 | 0.00 | 0.00 | 0.00 |
| Total | 15,187 | 15,187 | 17,965 | 17,965 | 8,336 | 8,336 | 9,596 | 9,596 | 11,166 | 11,166 | 12,039 | 12,039 |

**Table 16 Referral health care providers visit dataset**

| **Variables** | **Variable label in Bahasa Indonesia** | **Variable label in English** |
| --- | --- | --- |
| PSTV01 | Nomor peserta | Pseudo-anonymous ID members |
| PSTV02 | Nomor keluarga | Pseudo-anonymous Household ID |
| PSTV15 | Bobot | Weight |
| FKP02 | No Asal Rujukan | Primary care visit ID |
| FKL02 | ID Kunjungan | Visits ID |
| FKL03 | Tanggal datang kunjungan FKRTL | Admission date |
| FKL04 | Tanggal pulang kunjungan FKRTL | Discharge date |
| FKL05 | Provinsi FKRTL | Province of RHC |
| FKL06 | Kabupaten/Kota FKRTL | District of RHC |
| FKL07 | Kepemilikan FKRTL | Ownership of RHC |
| FKL08 | Jenis FKRTL | Type of RHC (Hospital, clinic) |
| FKL09 | Tipe FKRTL | Type of RHC (Hospital class, policlinic) |
| FKL10 | Tingkat Pelayanan FKRTL | Degree of services RHC (Inpatient, outpatient) |
| FKL11 | Jenis Poli FKRTL | Type of policlinics RHC |
| FKL12 | Segmen Peserta saat akses layanan FKRTL | Membership segmentation |
| FKL13 | Kelas iuran premi peserta saat akses layanan FKRTL | Inpatient ward class |
| FKL14 | Status pulang dari FKRTL | Discharge status |
| FKL15 | Diagnosis masuk pelayanan FKRTL (ICD-10 3 digit) | Diagnosis into RHC (ICD-10 3 digit) |
| FKL16 | Kode ICD 10 diagnosis masuk FKRTL (ICD-10 3-6 digit) | Code of diagnostic into RHC (ICD-10 3-6 digit) |
| FKL16A | Nama diagnosis masuk FKRTL (ICD-10 3-6 digit) | Name of diagnostic into RHC |
| FKL17 | Diagnosis primer pelayanan FKRTL (ICD-10 3digit) | Primary diagnosis name (ICD-10 3 digit) |
| FKL18 | Kode ICD 10 diagnosis primer FKRTL (ICD-10 3-6 digit) | Primary diagnostic code (ICD-10 3-6 digit) |
| FKL18A | Nama diagnosis primer FKRTL (ICD-10 3-6 digit) | Primary diagnosis name (ICD-10 3-6 digit) |
| FKL19 | Kode INACBGs | INACBGs code |
| FKL19A | Deskripsi kode INACBGs | INACBGs - Description of INACBGs code |
| FKL20 | INACBGs – Kode Casemix main groups (Digit ke-1) | INACBGs - Casemix main groups code (Digit 1) |
| FKL21 | INACBGs – Tipe kelompok kasus atau case groups | INACBGs - Casegroups |
| FKL22 | INACBGs – Spesifikasi kelompok kasus (Digit ke-3) | INACBGs - Specification of case groups (Digit 3) |
| FKL23 | INACBGs – Tingkat keparahan kelompok kasus(Digit | INACBGs - Degree of severity of case groups |
| FKL25 | Provinsi faskes perujuk | Province of PHC that made referral |
| FKL26 | Kabupaten/Kota faskes perujuk | District of PHC that made referral |
| FKL27 | Kepemilikan faskes perujuk | Ownership of PHC that made referral |
| FKL28 | Jenis faskes perujuk | Type of PHC that made referral |
| FKL29 | Tipe faskes perujuk | Type of PHC that made referral |
| FKL30 | Jenis prosedur | Type of procedure/treatment |
| FKL31 | Tarif regional INACBGs | Regional tariff |
| FKL32 | Group Tarif INACBGs | Group tariff |
| FKL33 | Kode special sub-acute groups (SA) | Code of special sub-acute groups |
| FKL34 | Tarif special sub-acute groups (SA) | Tariff of special sub-acute groups |
| FKL35 | Kode special procedures (SP) | Code of special procedure |
| FKL36 | Deskripsi special procedures (SP) | Description of special procedures |
| FKL37 | Tarif special procedures (SP) | Tariff of special procedures |
| FKL38 | Kode special prosthesis (RR) | Code of special prosthesis |
| FKL39 | Deskripsi special prosthesis (RR) | Description of special prosthesis |
| FKL40 | Tarif special prosthesis (RR) | Tariff of special prosthesis |
| FKL41 | Kode special investigation(SI) | Code of special investigation |
| FKL42 | Deskripsi special investigation(SI) | Description of special investigation |
| FKL43 | Tarif special investigation(SI) | Tariff of special investigation |
| FKL44 | Kode special drugs (SD) | Code of special drugs |
| FKL45 | Deskripsi special drugs (SD) | Description of special drugs |
| FKL46 | Tarif special drugs (SD) | Tariff of special drugs |
| FKL47 | Biaya Tagih – oleh fasilitas esehatan (provider) | The invoice cost by provider |
| FKL48 | Biaya Verifikasi - BPJS Kesehatan setelah dilakukan verifikasi | Verification cost by the BPJS |

**Table 17 Referral healthcare secondary diagnostic dataset**

| **Variable** | **Label in Indonesian** | **Label in English** |
| --- | --- | --- |
| FKL02 | ID kunjungan | Visit ID |
| FKL24 | Kode diagnosis | diagnosis code |
| FKL24_A | Kode dan nama diagnosis | code and name of diagnosis |

**Table 18 Referral healthcare visits by year**

| **Visit year** | **Frequency visits** | **Frequency individuals** | **Weighted frequency visits** | **Weighted frequency individuals** |
| --- | --- | --- | --- | --- |
| 2015 | 396,334 | 124,156 | 35,281,590 | 10,715,324 |
| 2016 | 511,421 | 151,359 | 42,629,071 | 12,468,313 |
| 2017 | 724,717 | 171,624 | 63,037,791 | 14,145,290 |
| 2018 | 874,087 | 192,532 | 76,175,728 | 16,132,319 |
| 2019 | 975,202 | 208,509 | 83,716,219 | 17,169,146 |
| 2020 | 786,423 | 167,733 | 65,009,669 | 13,337,415 |
| Total | 4,268,184 | 619,750 | 35,281,590 | 10,715,324 |

**Table 19 Referral healthcare: Most common primary diagnosis, 2015-2020**

| **ICD code** | **Name of diagnosis** | **Outpatient** | **Inpatient** | **Total visits** | **% to total RHC visits** |
| --- | --- | --- | --- | --- | --- |
| **All visits** | | 3,646,651 | 621,533 | 4,268,184 | 100% |
| Z00-Z99 | Factors influencing health status and visits to health services | 2,742,121 | 22,997 | 2,765,118 | 65% |
| K00-K95 | Diseases of the digestive system | 113,503 | 57,552 | 171,055 | 4% |
| H00-H95 | Diseases of the eye and adnexa; Diseases of the ear and mastoid process | 153,489 | 10,845 | 164,334 | 4% |
| O00-O9A | Pregnancy, childbirth and the puerperium | 18198 | 123530 | 141,728 | 3% |
| I00-I99 | Diseases of the circulatory system | 72,456 | 64,763 | 137,219 | 3% |
| J00-J99 | Diseases of the respiratory system | 82,923 | 46,018 | 128,941 | 3% |
| A00-B99 | Certain infectious and parasitic diseases | 36,096 | 84,084 | 120,180 | 3% |
| N00-N99 | Diseases of the genitourinary system | 76,041 | 39,325 | 115,366 | 3% |
| R00-R99 | Symptoms, signs and abnormal clinical and laboratory findings, not elsewhere classified | 87,502 | 19,619 | 107,121 | 3% |
| M00-M99 | Diseases of the musculoskeletal system and connective tissue | 61,423 | 10,810 | 72,233 | 2% |

Note: the diagnosis groups are ordered by total visits

**Table 20 Referral healthcare: Most common secondary diagnoses, 2005-2020**

| **ICD code** | **Name of Diagnosis** | **Number of visits** | **% Total** |
| --- | --- | --- | --- |
| Total visits | | 3,744,561 | 100% |
| I00-I99 | Diseases of the circulatory system | 681,208 | 18% |
| M00-M99 | Diseases of the musculoskeletal system and connective tissue | 412,273 | 11% |
| E00-E89 | Endocrine, nutritional and metabolic diseases | 350,828 | 9% |
| N00-N99 | Diseases of the genitourinary system | 330,441 | 9% |
| K00-K95 | Diseases of the digestive system | 273,224 | 7% |
| H00-H99 | Diseases of the eye and adnexa, and the ear and mastoid process | 208,234 | 6% |
| J00-J99 | Diseases of the respiratory system | 196,061 | 5% |
| O00-O9A | Pregnancy, childbirth and the puerperium | 179,649 | 5% |
| G00-G99 | Diseases of the nervous system | 177,781 | 5% |
| Z00-Z99 | Factors influencing health status and visits to health services | 134,144 | 4% |

Note:

A secondary diagnosis is a diagnosis made in conjunction with the primary diagnosis when a patient or individual is admitted to or during treatment. Secondary diagnoses can include comorbidities and/or complications.

## Figures

**Figure 1 Revision to the Presidential Regulation on National Health Insurance 2013-2024**

**Figure 2 Rate of sample-weighted individuals who visited PHC in 2015-2020 by 10,000 BPJS-K members in quartile groups**

| 2015 |
| --- |
| Mean: 3702.288  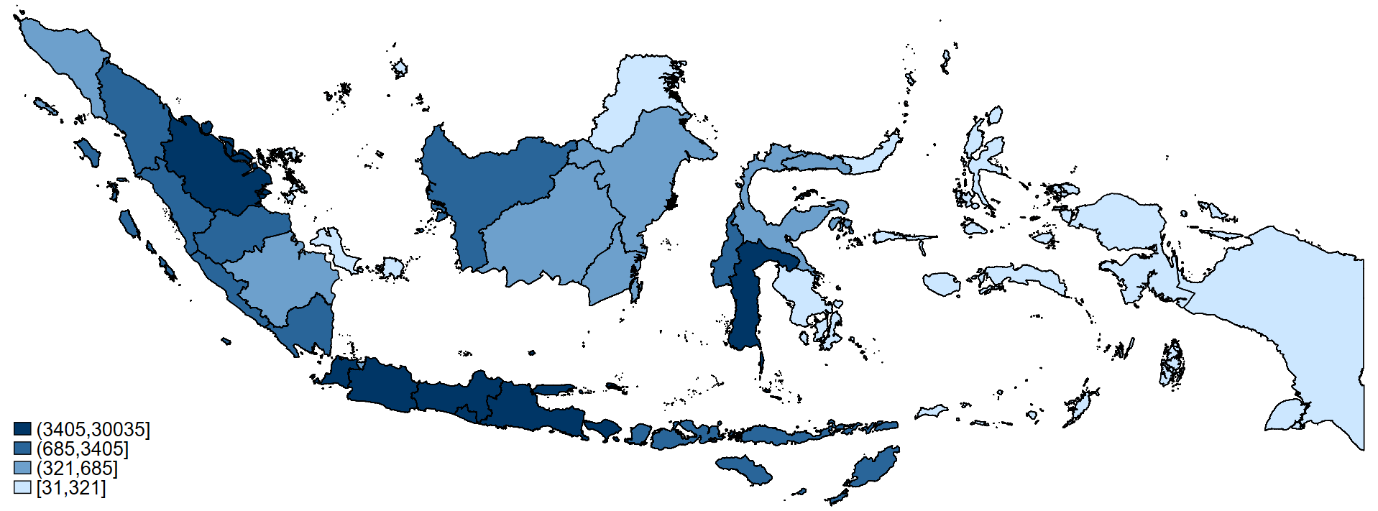 |
| 2016 |
| Mean: 4417.628  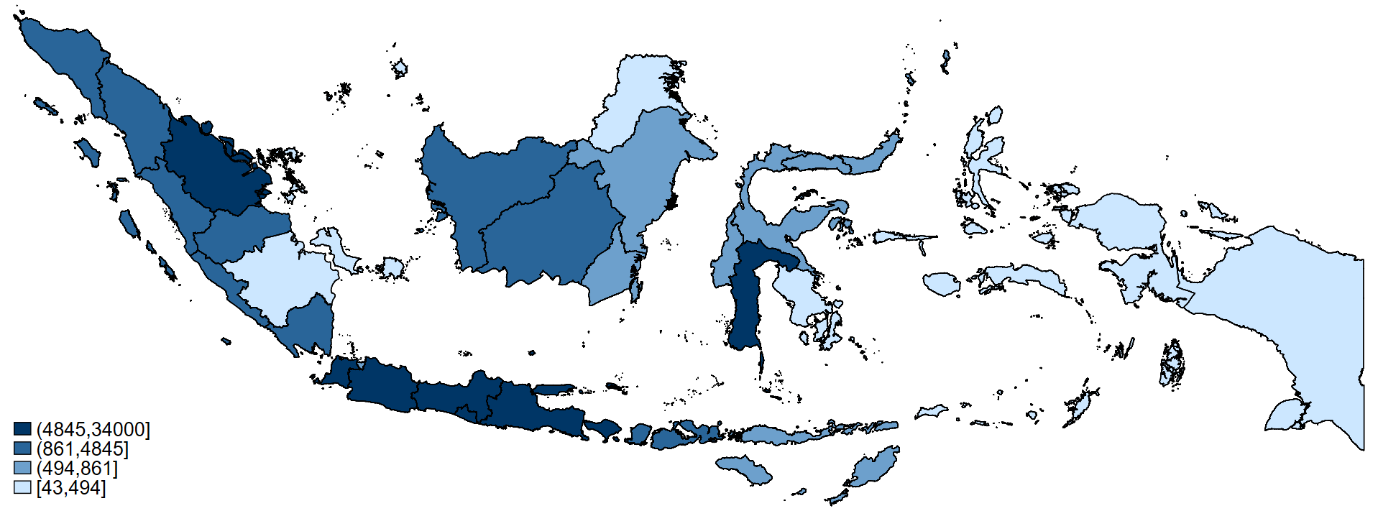 |
| 2017 |
| Mean: 7884.356  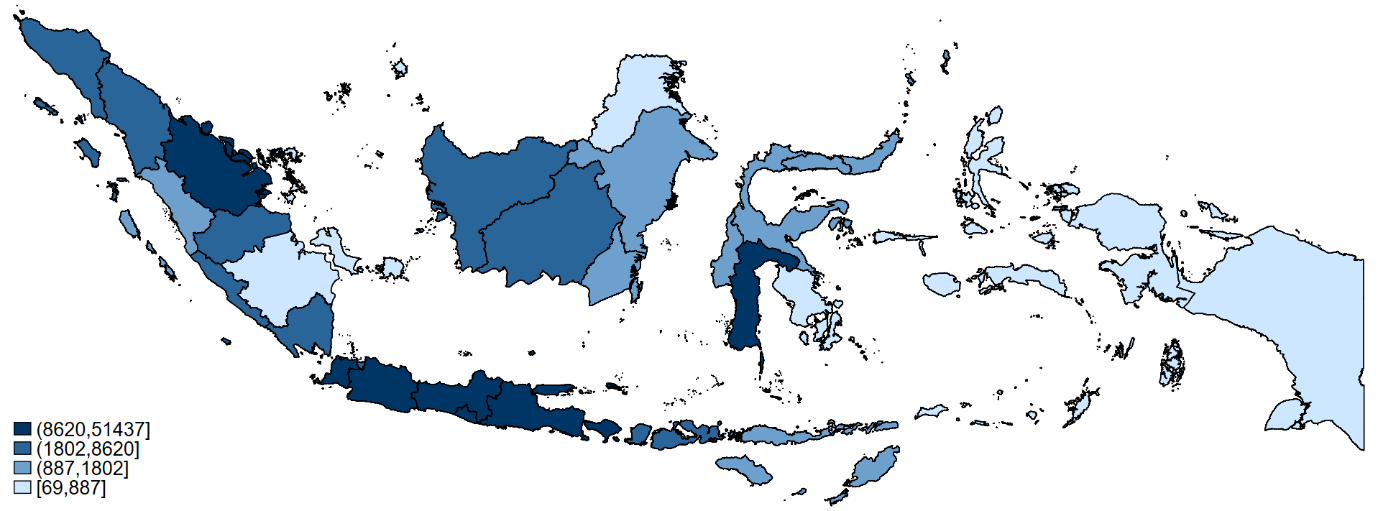 |
| 2018 |
| Mean: 7864.597  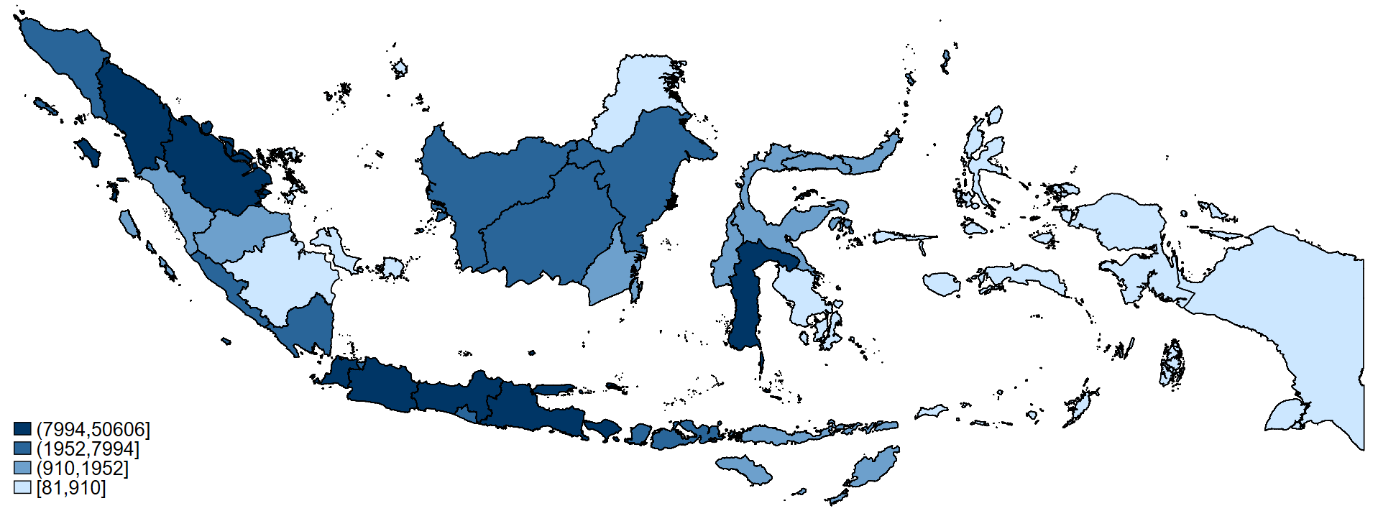 |
| 2019 |
| Mean: 8074.507  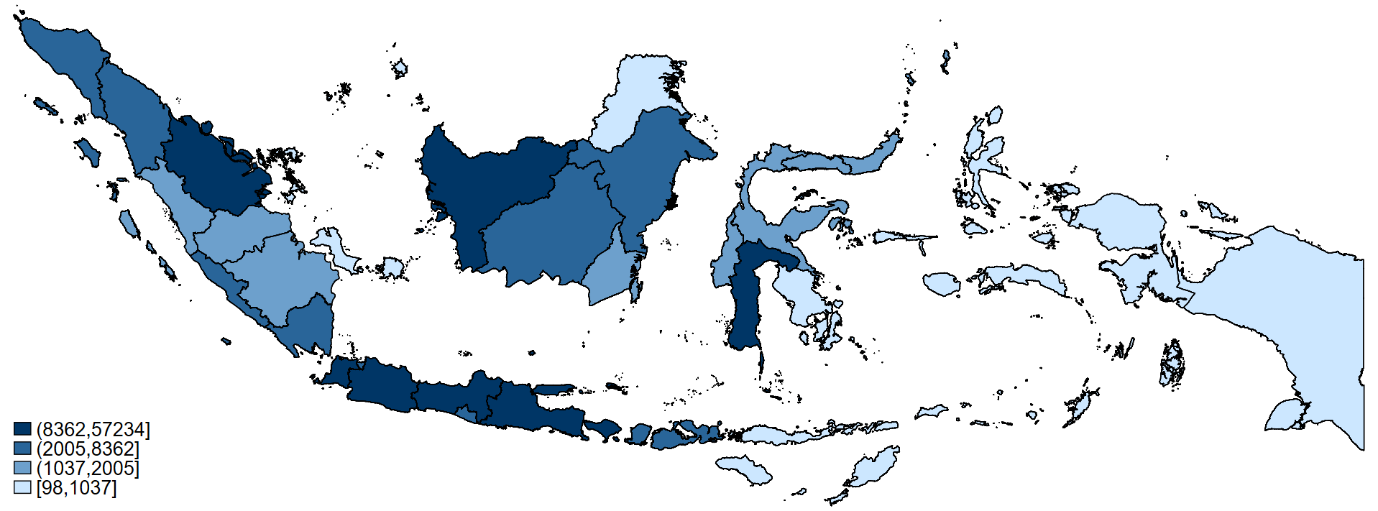 |
| 2020 |
| Mean: 2512.354  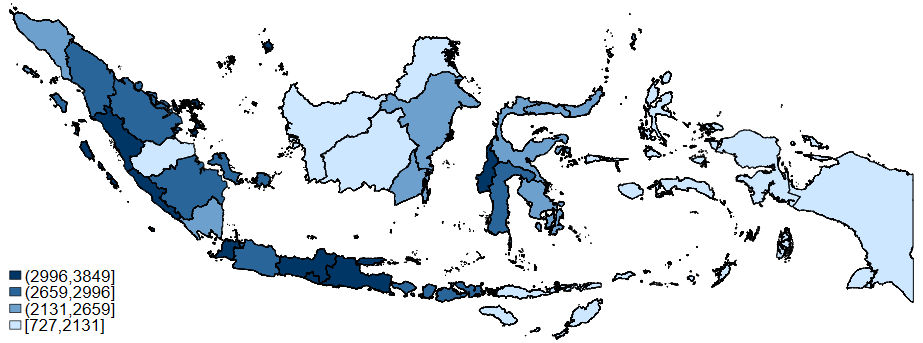 |
| Source: Authors’ calculation. The BPJS-K registered members by province are from the National Insurance Board ([DJSN](https://kesehatan.djsn.go.id/kesehatan/cakupan/)) website.  Notes: The black line is the provinces’ border |

**Figure 3 Number of diagnoses based on the number of primary healthcare visits per diagnosis for services under capitation, 2015-2020**

**Figure 4 Most common diagnosis of primary healthcare visits for services under capitation, 2015 – 2020**

3%

Note: X-axis is number of visits. The percentage shows a proportion of visits with certain diagnosis to the total visits made during 2015-2020.

**Figure 5 Referral healthcare: Number of primary diagnosis based on the number of visits per diagnosis, 2015-2020**

**References**

1. Sufriyana H, Wu YW, Su ECY. Artificial intelligence-assisted prediction of preeclampsia: Development and external validation of a nationwide health insurance dataset of the BPJS Kesehatan in Indonesia. EBioMedicine. 2020;54.

2. Husnayain A, Fuad A, Laksono IS, Su ECY. Improving dengue surveillance system with administrative claim data in Indonesia: Opportunities and challenges. Studies in Health Technology and Informatics. 2020;270(172):853–7.

3. Husnayain A, Ekadinata N, Sulistiawan D, Su ECY. Multimorbidity patterns of chronic diseases among indonesians: Insights from indonesian national health insurance (inhi) sample data. International Journal of Environmental Research and Public Health. 2020;17(23):1–12.

4. Handayani PW, Dartanto T, Moeis FR, Pinem AA, Azzahro F, Hidayanto AN, et al. The Regional and referral compliance of online healthcare systems by Indonesia National Health Insurance Agency and health-seeking behavior In Indonesia. Heliyon [Internet]. 2021;7(9):e08068. Available from: https://doi.org/10.1016/j.heliyon.2021.e08068

5. Schaefers J, Wenang S, Afdal A, Mukti AG, Sundari S, Haier J. Population-based study on coverage and healthcare processes for cancer during implementation of national healthcare insurance in Indonesia. The Lancet Regional Health - Southeast Asia [Internet]. 2022;6:100045. Available from: https://doi.org/10.1016/j.lansea.2022.100045

6. Sambodo NP, Bonfrer I, Sparrow R, Pradhan M, van Doorslaer E. Effects of performance-based capitation payment on the use of public primary health care services in Indonesia. Social Science and Medicine [Internet]. 2023;327(April):115921. Available from: https://doi.org/10.1016/j.socscimed.2023.115921

7. Fritz M. Temperature and non-communicable diseases: Evidence from Indonesia’s primary health care system. Health Economics (United Kingdom). 2022;31(11):2445–64.

8. Gultom EI, Afriandi I, Gondodiputro S. Perbedaan utilitas pasien DM di FKTP sebelum dan sesudah penerapan kebijakan Kapitasi Berbasis Kinerja (KBK) di Kota Cimahi (Analisis data sampel BPJS 2015-2020). Jurnal Kebijakan Kesehatan Indonesia. 2023;12(1):15–21.

9. Dewi E, Sofiatin Y, Setiawati E, Wahyudi K, Afriandi I. Analisis pembiayaan JKN pasien hipertensi di FKTP Jawa Barat tahun 2015-2016. Jurnal Kebijakan Kesehatan Indonesia. 2021;10(02):78–85.

10. Fitrilia I. Pemanfaatan pelayanan hemodialisis pada peserta Jaminan Kesehatan Nasional (Analisis data sampel BPJS Tahun 2015-2016) [Internet]. Universitas Gadjah Mada; 2021. Available from: http://etd.repository.ugm.ac.id/penelitian/detail/203667

11. Fitrian W, Sofiatin Y, Afriandi I. Pola pelayanan penderita hipertensi peserta JKN di FKRTL Provinsi Jawa Barat tahun 2015-2016. Jurnal Kebijakan Kesehatan Indonesia [Internet]. 2021;10(03):143–50. Available from: https://journal.ugm.ac.id/jkki/article/view/64161

12. Setiawan E, Nurjannah N, Komaryani K, Nugraha RR, Thabrany H, Purwaningrum F, et al. Utilization patterns of healthcare facility and estimated expenditure of PLHIV care under the Indonesian National Health Insurance Scheme in 2018. BMC Health Services Research [Internet]. 2022;22(1):1–9. Available from: https://doi.org/10.1186/s12913-021-07434-9

13. Ng JYS, Ramadani RV, Hendrawan D, Duc DT, Kiet PHT. National Health Insurance Databases in Indonesia, Vietnam and the Philippines. PharmacoEconomics - Open [Internet]. 2019;3(4):517–26. Available from: https://doi.org/10.1007/s41669-019-0127-2

14. Nugroho ST, Ahsan A, Kusuma D, Adani N, Irawaty DK, Amalia N, et al. Income Disparity and Healthcare Utilization: Lessons from Indonesia’s National Health Insurance Claim Data. Asian Pacific Journal of Cancer Prevention. 2023;24(10):3397–402.

15. Rachmawaty R, Sinrang AW, Wahyudin E, Bukhari A. Evaluation of health care quality among insured patients in Indonesian mother & child hospital: A secondary data analysis. Gaceta Sanitaria [Internet]. 2021;35:S613–8. Available from: https://doi.org/10.1016/j.gaceta.2021.10.100

16. Sambodo NP, Van Doorslaer E, Pradhan M, Sparrow R. Does geographic spending variation exacerbate healthcare benefit inequality? A benefit incidence analysis for Indonesia. Health Policy and Planning [Internet]. 2021 Aug 1;36(7):1129–39. Available from: https://doi.org/10.1093/heapol/czab015

17. Chalkley M, Hidayat B, Ramadani RV, Aragón MJ. The sensitivity of hospital coding to prices: evidence from Indonesia. International journal of health economics and management. 2022 Jun;22(2):147–62.

18. BPJS Kesehatan. Data Sample BPJS Kesehatan Tahun 2015-2016 edisi revisi. Vol. 19. 2019. 1–53 p.

19. BPJS Kesehatan. Buku Data Sampel 2015-2020. 2021.

20. BPJS Kesehatan. Data Sampel BPJS Kesehatan 2015-2018. 2020.
